# Self‐Assembly of Cholane–Phenanthrene–Cholane Trimers for Light‐Harvesting Supramolecular Systems

**DOI:** 10.1002/cbic.202500121

**Published:** 2025-04-10

**Authors:** Edouard Ehret, Ioan Iacovache, Simon M. Langenegger, Benoît Zuber, Robert Häner

**Affiliations:** ^1^ Department of Chemistry, Biochemistry, Pharmaceutical Sciences University of Bern Freiestrasse 3 CH‐3012 Bern Switzerland; ^2^ Institute for Anatomy University of Bern Baltzerstrasse 2 CH‐3012 Bern Switzerland

**Keywords:** amphiphiles, energy transfer, light‐harvesting complex, phosphodiester, supramolecular polymers

## Abstract

The self‐assembly of two isomeric amphiphilic cholane–phenanthrene–cholane trimers and the light‐harvesting properties of the formed supramolecular assemblies in aqueous medium are presented. Distinct differences in the supramolecular nanostructures are observed based on the type of phenanthrene isomer used, as well as on the cooling rate applied during the thermal self‐assembly process. A fast cooling rate results in the formation of 2D objects (nanosheets) with both trimers. In contrast, a slow cooling process results in the observation of 3D objects, with worm‐like nanostructures for the self‐assembled 3,6‐disubstituted phenanthrene trimer and nanotubes for the 2,7‐isomer. Upon doping with an acceptor chromophore, the supramolecular phenanthrene assemblies demonstrate efficient energy transfer. The presence of small quantities (6%) of a pyrene acceptor results in a fivefold increase in the quantum yield compared to the phenanthrene trimer alone, highlighting their potential as artificial light‐harvesting complexes. This work underscores the versatility of amphiphilic phenanthrene derivatives as building blocks for light‐harvesting supramolecular systems.

## Graphical abstract

The self‐assembly of phosphodiester‐linked cholane–phenanthrene–cholane trimers is described. The morphology of the formed nanostructures depends on the phenanthrene isomer used and on the cooling rate during the assembly process leading to nanosheets, worm‐like structures, or nanotubes. Doping with pyrene results in a strong increase in the quantum yield, demonstrating the potential of these assemblies as artificial light‐harvesting systems.

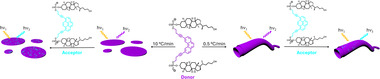

## 1 Introduction

The development of self‐assembling supramolecular systems represents a cornerstone of modern materials science, offering unprecedented opportunities to design functional nanomaterials.^[^ ^1^, ^2^, ^3^, ^4^, ^5^, ^6^, ^7^, ^8^ ^]^ These materials are composed of repeating molecular units held together by noncovalent interactions, enabling precise structural and functional control while retaining dynamic reversibility.^[^ ^9^, ^10^, ^11^, ^12^, ^13^, ^14^, ^15^, ^16^, ^17^ ^]^ Among the various approaches, amphiphilic, π‐conjugated building blocks have proven particularly effective for the assembly of nanostructures with tailored properties for applications ranging from drug delivery to optoelectronics and light‐harvesting complexes (LHCs).^[^ ^18^, ^19^, ^20^, ^21^, ^22^, ^23^, ^24^, ^25^, ^26^, ^27^, ^28^ ^]^ In LHCs, efficient energy transfer depends on the ordered positioning of chromophores, which self‐assembly facilitates by allowing precise spatial arrangements and fine‐tuning of their organization.^[^ ^29^, ^30^, ^31^ ^]^ Moreover, the ability to modify the individual building block with different chromophores makes supramolecular polymers (SPs) ideal candidates for energy transfer systems, light‐harvesting devices, or advanced sensing technologies. Previously, we reported on the self‐assembly of amphiphilic, π‐conjugated, phosphodiester‐linked trimers in aqueous media.^[^ ^32^, ^33^, ^34^ ^]^ Upon a controlled cooling step, these compounds were shown to form supramolecular assemblies with various morphologies ranging from nanosheets to nanotubes and nanoribbons. Additionally, such assemblies were shown to possess impressive light‐harvesting properties.^[^ ^35^, ^36^ ^]^

**Figure 1:**
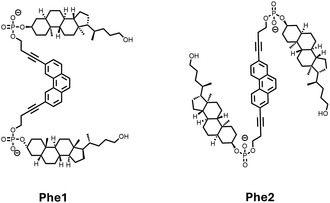
The two cholane‐phenanthrene‐cholane trimers **Phe1** and **Phe2** used in this study.

In this work, we report the synthesis of two phosphodiester‐linked cholane–phenanthrene–cholane trimers bearing two different substitution patterns (**Phe1** and **Phe2**, **Figure** **1**) and their self‐assembly, as well as light‐harvesting properties in the presence of a suitable acceptor chromophore. While the phenanthrene units act as chromophores, the hydrophobic cholanes promote self‐assembly in an aqueous environment. Doping of the self‐assembled nanostructures with a pyrene chromophore resulted in an efficient energy transfer process.

## 2 Results and Discussion

### 2.1 Supramolecular Assembly of Cholane–Phenanthrene–Cholane Trimers into Different Types of Morphologies

The synthesis of **Phe1** and **Phe2** was performed by adapting published procedures.^[^ ^33^, ^35^ ^]^ Lithocholic acid was reduced to its diol form and selectively protected. Subsequently, it was coupled to its phenanthrene diphosphoramidite. The trimers were deprotected, purified by RP‐HPLC, and characterised by HRMS (see Supporting Information).

**Figure 2:**
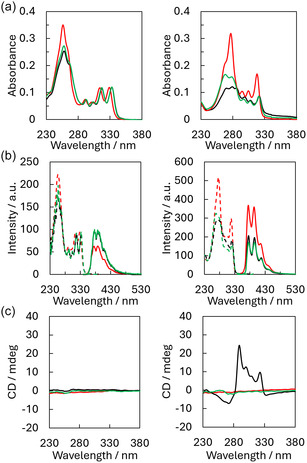
Spectroscopic characterization of **Phe1** (left) and **Phe2** (right). The spectra were measured at 70 °C (red) and 20 °C, after slow cooling (black) and after fast cooling (green). a) Temperature‐dependent UV‐vis absorption spectra; b) fluorescence excitation spectra (dotted line) and fluorescence emission spectra (solid line) left: λ_ex_.: 333 nm and λ_em_.: 375 nm, right: λ_ex_.: 320 nm and λ_em_.: 394 nm; c) CD spectra. Conditions: 3 μM oligomer, 10 mM sodium phosphate buffer pH 7.2, 10 mM NaCl, and EtOH (15% for **Phe1** and 20% for **Phe2**).

Temperature‐dependent UV‐vis absorption spectra were recorded for both trimers to investigate their self‐assembly process (**Figure** **2**a). To ensure complete disassembly, all samples were initially heated to 70 °C and then cooled to 20 °C with two different rates: 0.5 °C min^−1^ (slow) and 10 °C min^−1^ (fast). During the cooling process (70–20 °C), characteristic changes were observed in the absorbance of the two trimers, indicating the self‐assembly of the SPs. For **Phe1**, the spectrum showed a bathochromic shift for the bands between 314 and 328 nm accompanied by a hypochromic effect at 257 nm at 20 °C after applying the slow cooling gradient (black curve). Similarly, for **Phe2**, a pronounced hypochromicity was evident across the spectrum, along with a 3 nm red shift for bands between 294 and 318 nm. The self‐assembly process of both trimers followed a nucleation‐elongation process which starts around 51 °C (Figure S18, Supporting Information).^[^ ^14^, ^37^ ^]^ The change to a faster cooling rate did not have a significant influence on the absorption by the self‐assembly formed by **Phe1** at 20 °C (green curve). It did, however, induce changes in the absorption by the self‐assembled **Phe2**, as observed by the smaller hypochromic effect present in the region between 260 nm and 290 nm. Further characterization of the assemblies involved measuring temperature‐dependent excitation and emission fluorescence spectra (Figure 2b). Excitation of **Phe1** at 333 nm revealed an increase in the phenanthrene monomer emission upon self‐assembly regardless of the temperature gradient applied. In contrast, **Phe2** exhibited a strong decrease in phenanthrene monomer fluorescence upon cooling, with a more pronounced reduction under the fast cooling gradient. Furthermore, circular dichroism (CD) spectra were recorded before and after the self‐assembly process (Figure 2c). In its assembled state, **Phe1** did not show any CD signal after either cooling condition. However, **Phe2** displayed dichroic signals after self‐assembly using the slow cooling gradient, as indicated by the black curve. This effect was absent when the fast cooling gradient was applied. The changes observed in the UV‐vis and fluorescence spectra suggest that an aggregation process is occurring for both trimers and that this process differs for **Phe2** depending on the cooling rate applied.

**Figure 3:**
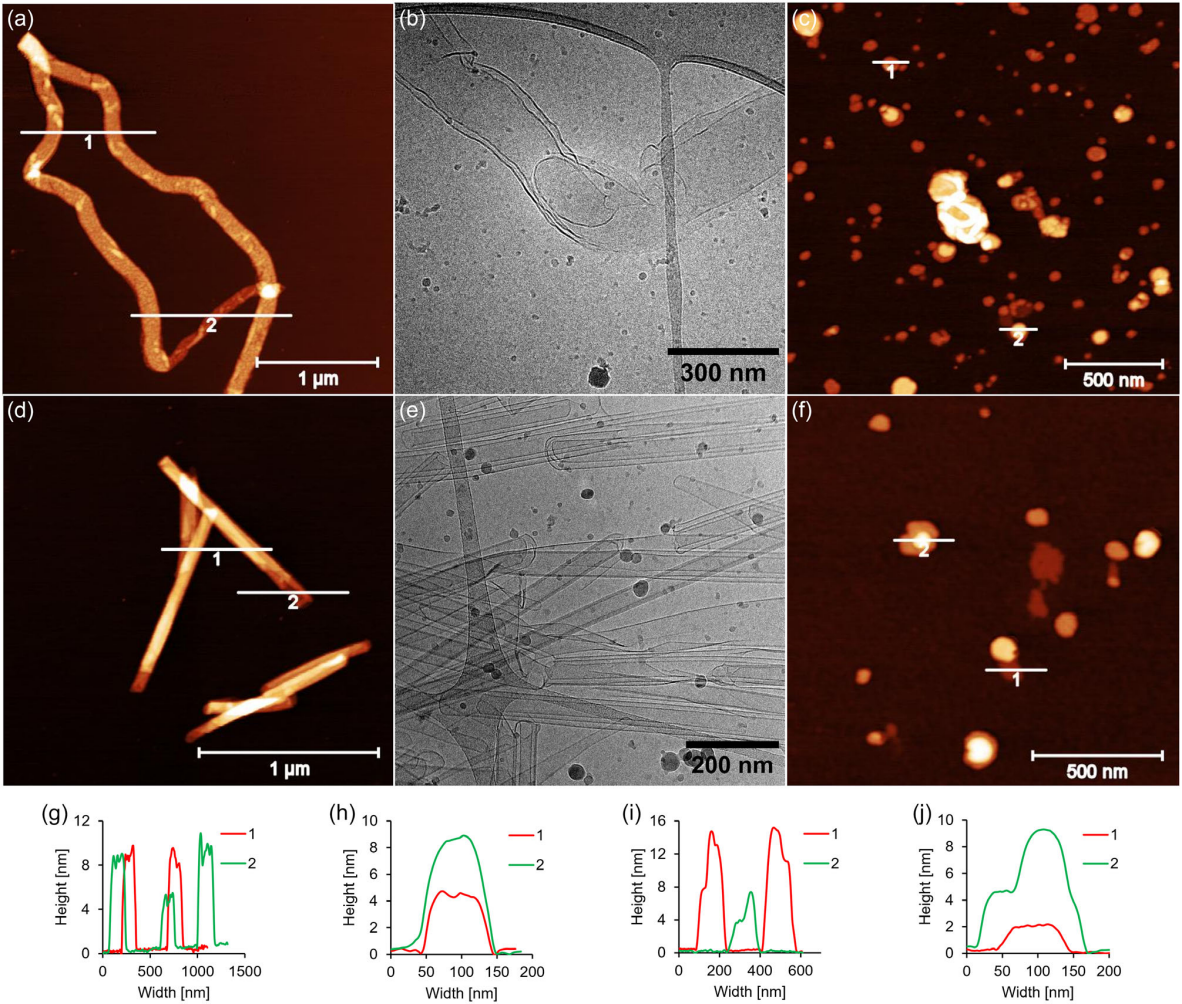
Microscopy measurements of oligomers **Phe1** (top) and **Phe2** (bottom). AFM images of the oligomers after a cooling rate of a,d) 0.5 °C min^−1^; c,f) 10 °C min^−1^; b,e) cryo‐EM images of the oligomers after cooling (0.5 °C min^−1^); g–j) cross sections of the AFM images a, c, d, and f, respectively. Conditions: 3 μM oligomer, 10 mM sodium phosphate buffer pH 7.2, 10 mM NaCl, and EtOH (15% for **Phe1**; 20% for **Phe2**).

The morphologies of the self‐assembled aggregates were studied by atomic force microscopy (AFM) and cryo‐electron microscopy (cryo‐EM, **Figures** **3** and S21–S26, Supporting Information). After assembly, the samples were deposited onto either (3‐aminopropyl)triethoxysilane (APTES)‐modified mica sheets for AFM or on copper lacey carbon grids for cryo‐EM. Despite exhibiting identical spectroscopic properties under two different cooling gradients, **Phe1** self‐assembled into two distinct supramolecular nanostructures depending on the cooling rate. AFM and cryo‐EM measurements revealed worm‐like nanostructures formed at a slow cooling gradient (Figure 3a,b), while circular nanosheets were observed at a fast cooling rate (Figure 3c). Cryo‐EM images show that the worm‐like structures are between 100 and 310 nm wide, up to 30 μm long, and possess a double membrane. AFM cross sections indicate heights of 4 to 8 nm suggesting a membrane thickness of ≈2 nm. The observed nanosheets have a height of 4 or 8 nm and possess a diameter varying from 50 to 150 nm. In contrast, **Phe2**, the 2,7‐disubstituted trimer, self‐assembled into nanotubes at a slow cooling rate (Figure 3d,e) and into nanosheets at a fast rate (Figure 3f). Cryo‐EM measurements show the presence of single‐ and multi‐walled nanotubes, with widths ranging from 30 to 80 nm and up to 2 μm long. AFM imaging measurements correlate well with these findings, showing nanotube heights ranging from 4 to 16 nm, with 4 nm increments. This indicates a membrane thickness of about 2 nm. The nanosheets formed by this phenanthrene isomer were thinner than those formed by **Phe1**, with a height of 2 nm and a diameter ranging from 50 to 200 nm. Thus, in addition to the molecular structure of the phenanthrene units, the assembly process is also significantly affected by the cooling rate.^[^ ^38^ ^]^

### 2.2 Light‐Harvesting Properties of **Phe1**

**Figure 4:**
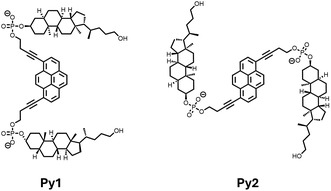
Chemical structures of both acceptor oligomers used as doping agents.

**Figure 5:**
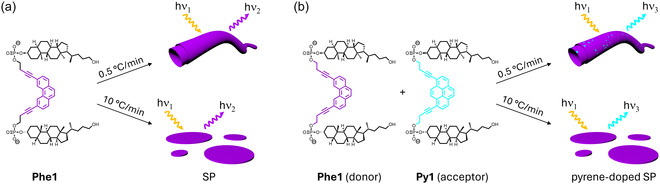
Illustration of an idealized assembly of an SP of **Phe1** (purple) in the absence a) or presence b) of an acceptor oligomer (blue), which is randomly integrated into the forming SP. For clarity, only one acceptor oligomer (**Py1**) is indicated here, but equal results are obtained with **Py2**.

Next, we studied the light‐harvesting properties of the **Phe1**‐based SPs. For this purpose, the assembly of **Phe1** was performed in the presence of small quantities of a cholane–pyrene–cholane trimer (**Py1** or **Py2**, **Figure** **4**). The pyrene‐containing trimers are incorporated into the growing SPs during the assembly process and can act as acceptor chromophores after excitation of phenanthrene.^[^ ^39^ ^]^ Thus, pyrene‐doped SPs were assembled following different temperature gradients (0.5 and 10 °C min^−1^) as described above. An illustration of the formation of the SP via self‐assembly of **Phe1** in the absence of **Py1** is shown in **Figure** **5**a, as well as in the presence of **Py1** in Figure 5b. Similar results were obtained in light‐harvesting experiments using **Phe2**. For simplicity, only the data acquired with **Phe1** are described.

**Figure 6:**
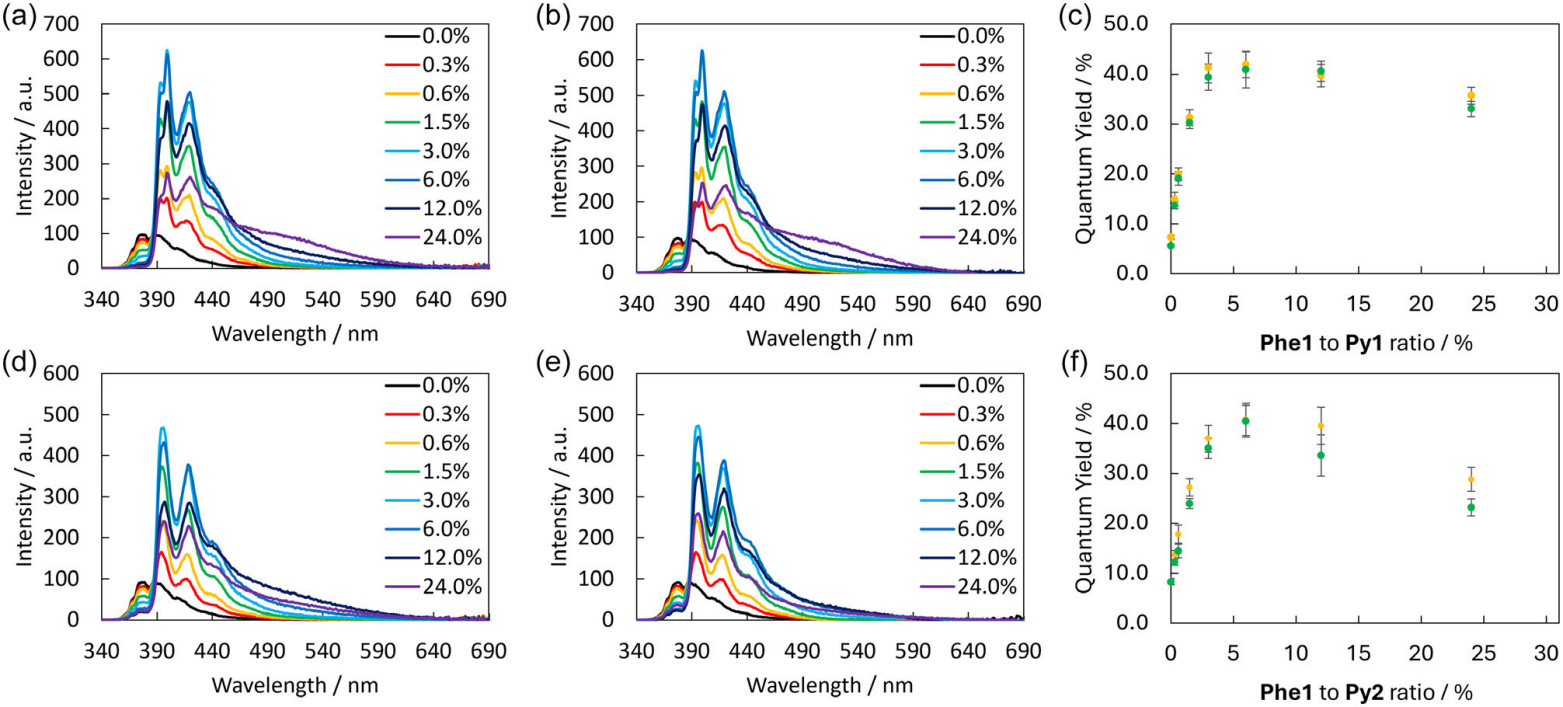
Fluorescence emission spectra of **Phe1** in the presence of increasing amounts of **Py1** (top) and **Py2** (bottom) (0 to 24%) with a cooling rate of a,d) 0.5 °C min^−1^ and b,e) 10 °C min^−1^. c,f) Quantum yield of both systems, in green at 0.5 °C min^−1^ and in yellow at 10 °C min^−1^. Conditions: 3 μM **Phe1**, 10 mM sodium phosphate buffer pH 7.2, 10 mM NaCl, and 15% EtOH, λ_ex_: 333 nm.

The light‐harvesting properties of the supramolecular nanostructures formed by **Phe1** were determined by recording fluorescence spectra (**Figure** **6**) in the presence of up to 24% of **Py1** (top) or **Py2** (bottom). Selective excitation of **Phe1** at 333 nm leads to major changes in the fluorescence emission spectrum of the system at 20 °C after slow and fast cooling rates (Figure 6a,b, respectively). The gradual increase in the amount of acceptor chromophore leads to a decrease in phenanthrene emission (max. at 376 nm, black line) while the pyrene monomer emission (four maxima at 393, 399, 419 and 440 nm) reaches maximum intensity after the addition of 6% of **Py1** before decreasing again. In addition, the addition of larger quantities of **Py1** (24%) leads to the appearance of a broad band (from 460 to 580 nm). This band is attributed to pyrene excimer formation, since rising concentrations of pyrene molecules increase the probability of molecular interaction.^[^ ^40^ ^]^ These changes are in correlation with the fluorescence quantum yield (Φ_F_, Figure 6c) of the system, which increases from 8.3 ± 0.6% to a maximum of 41.9% ± 2.6% at 6% of **Py1,** followed by a slight decrease. The cooling rate applied to the system did not have a significant effect on the fluorescence properties as can be seen in Figure 6c. The quantum yields are equal (**Table** **1**) for SPs obtained by applying either of the temperature gradients. Similar results were obtained after the addition of **Py2** with the appearance of pyrene monomer emission (three maxima at 396, 418 and 440 nm) after slow and fast cooling rates (Figure 6d,e, respectively). Also in this case, the addition of increasing amounts of **Py2** results in a decrease in the phenanthrene emission and an increase in the pyrene monomer emission. A maximum in fluorescence emission is reached after the addition of 6% of **Py2**, while a distinct pyrene excimer emission band is present after the addition of 12–24% of the acceptor. The fluorescence quantum yield (Figure 6f) reaches a maximum of 40.4 ± 3.2% after the addition of 6% of **Py2** (Table 1), which corresponds to a fivefold increase compared to the system in the absence of an acceptor. AFM measurements performed on doped systems in the presence of 6% of the acceptor trimer (see Figures S37 and S38, Supporting Information) revealed that the presence of the acceptor did not influence the morphology of the different SPs.

**Table 1:** Fluorescence quantum yields after incorporation of pyrene acceptors into Phe1 SPs.

Acceptor	Temperature gradient [°C min^−1^]	Quantum yield [%]a)	Increase in Φ_F_ b)
**Py1**	0.5	41.9 ± 2.6	5×
	10	40.9 ± 3.7	5×
**Py2**	0.5	40.4 ± 3.2	5×
	10	40.8 ± 3.2	5×

a) At a 6 mol‐% acceptor/donor ratio.

b) Compared to Φ_F_ of **Phe1** without doping agent: 8.3 ± 0.6%.

The observations described above indicate that an energy transfer process takes place between phenanthrene and pyrene molecules. The decrease in fluorescence of the donor (phenanthrene) in the presence of the acceptor (pyrene) can be explained by a FRET mechanism.^[^ ^41^, ^42^ ^]^ However, the increase in the quantum yields indicates that additional energy transfer mechanisms are also involved (e.g., coherent energy transfer).^[^ ^43^, ^44^, ^45^ ^]^

Interestingly, the difference in the supramolecular morphology did not have any influence on the light‐harvesting capabilities of the **Phe1** donor trimer as highlighted by the nearly identical quantum yields observed after both cooling rates were applied (see Table 1).

## 3 Conclusions

The synthesis and self‐assembly processes of two isomeric phosphodiester‐linked cholane–phenanthrene–cholane trimers have been described. AFM and cryo‐EM measurements revealed different types of morphologies depending on the phenanthrene isomer used and the cooling gradient applied during a thermally controlled self‐assembly process. For **Phe1**, a cooling rate of 0.5 °C min^−1^ resulted in the formation of worm‐like nanostructures. Yet, applying a cooling rate of 10 °C min^−1^ resulted in the formation of supramolecular nanosheets. Furthermore, the second isomer investigated, **Phe2**, self‐assembled into single‐ and multi‐walled nanotubes when a cooling rate of 0.5 °C min^−1^ was applied. Finally, using a faster cooling gradient (10 °C min^−1^), the self‐assembly of **Phe2** resulted in the formation of nanosheets.

In addition, the supramolecular assemblies of the 3,6‐dialkynyl phenanthrene trimer (**Phe1**) revealed remarkable light‐harvesting properties. Doping of the supramolecular assembly with a pyrene acceptor chromophore leads to an efficient energy transfer between phenanthrenes and the pyrenes, regardless of the morphology (worm‐like nanostructure or nanosheet). Thus, the addition of 6% of **Py1** or **Py2** resulted in a fluorescence quantum yield of 40%, which corresponds to a fivefold increase compared to the native (undoped) **Phe1**. These data demonstrate that the supramolecular assembly of short, amphiphilic oligomers has a positive effect on the energy transfer processes between the phenanthrene and pyrene chromophores. In view of the simplicity of the assembly process and their compatibility with aqueous conditions, cholane–phenanthrene–cholane phosphodiester‐linked compounds present promising building blocks for artificial LHCs and other functional supramolecular systems.

## Conflict of Interest

The authors declare no conflict of interest.

## Supporting information

Supplementary Material

## Data Availability

The data that support the findings of this study are available in the supplementary material of this article.
