# Supplementary material for: Self‐Assembly of Cholane–Phenanthrene–Cholane Trimers for Light‐Harvesting Supramolecular Systems
Source: Chembiochem. 2025 Apr 10;26(12):e202500121. doi: 10.1002/cbic.202500121 (PMC12177694; doi:10.1002/cbic.202500121)
Supplement: Supplementary file 1 — Supplementary Material [file CBIC-26-e202500121-s001.pdf]

Electronic Supplementary Information

Self-Assembly of Cholane-Phenanthrene-Cholane Trimers for Light-Harvesting Supramolecular Systems

Edouard Ehret,[a] Ioan Iacovache,[b] Simon M. Langenegger,[a] Benoît Zuber,[b] and Robert Häner[a]*

[a] Department of Chemistry, Biochemistry, and Pharmaceutical Sciences, University of Bern, Freiestrasse 3, CH-3012 Bern, Switzerland

[b] Institute of Anatomy, University of Bern, Baltzerstrasse 2, CH – 3012 Bern, Switzerland

Table of contents

1. General Methods 3

2. Synthesis of the Trimers 4

2.1. Organic Synthesis 4

2.2. NMR Spectra 8

2.3. MS Spectra 11

2.4. HPLC Trace 14

3. Preparation of the samples 14

4. Temperature-dependent UV-vis Spectra 14

5. Atomic Force Microscopy 15

6. Cryo-EM 17

7. Quantum Yield Calculations 18

8. Light-Harvesting Spectroscopic Data 24

9. References 29

1. General Methods

All reagents and solvents were purchased from commercial sources and used without further purification. Water was used from a Milli-Q system. Mass spectra were measured by the Analytical Research and Services (ARS) of the University of Bern, Switzerland, on a Thermo Fisher LTQ Orbitrap XL using Nano Electrospray Ionization (NSI). Mass spectra were measured in positive and negative ion modes in acetonitrile/water/triethylamine mixtures. UV-Vis spectra were measured on a Jasco V-730 spectrophotometer using quartz cuvettes with an optical path of 1 cm. Fluorescence spectra were collected on a Jasco spectrophotometer FP-8300 using an excitation slit of 1 nm and an emission slit of 2.5 nm. CD measurements were performed on a Jasco J-715 spectropolarimeter equipped with a Jasco PS-150J power supply and a Jasco PFD-350S thermostat. Supramolecular self-assembly was carried out via thermal disassembly and reassembly. The sample solution was heated to 70 °C, then cooled with a gradient of 0.5 °C/min (or 10 °C/min) to 20 °C in a thermostat equipped with a Peltier. Atomic force microscopy (AFM) experiments were conducted under ambient conditions on a Nanosurf FlexAFM instrument using tapping mode. AFM samples were prepared on (3-aminopropyl)triethoxysilane (APTES)-modified mica sheets (Glimmer "V1", 20 mm x 20 mm, G250-7, Plano GmbH) according to published procedures.[1] The mica sheets were freshly cleaved and mounted with tape on top of a desiccator (3 L) before the desiccator was purged with argon. APTES (30 μL) was pipetted into an Eppendorf tube cap and Hünig's base (10 μL) was added into a second cap. Both Eppendorf tube caps were placed at the bottom of the desiccator below the mica sheets, then the desiccator was closed. The mica sheets were left for one night in the desiccator to cure. Afterwards, the corresponding sample solution (20 μL) was pipetted onto the APTES-modified mica sheet. After an adsorption time of 10 min, the mica sheet was rinsed with Milli-Q water (2 mL) and dried under a stream of argon. Samples for cryo-EM were plunge-frozen using the FEI Vitrobot Mark 4 at room temperature and 100% humidity. In brief, copper lacey carbon grids were glow discharged (air -10 mA for 20 seconds). 3 μL of the sample were pipetted on the grids and blotted for 3 seconds before plunging into liquid ethane. Sample grids were stored in liquid nitrogen. Images were acquired using a Gatan 626 cryo holder on a Falcon III equipped FEI Tecnai F20 in nanoprobe mode. Due to the nature of the sample, acquisition settings had to be adjusted for a low total electron dose (less than 20 $e^-/Å^2$) using EPU software. Distance measurements were done with Fiji[2,3] using the multi-point tool to set marks. The ethanol content in the buffer medium was adjusted for each trimer individually to ensure an experimentally workable temperature window (i.e., completely de-aggregated form at 70 °C and fully aggregated form at 20 °C).

2. Synthesis of the Trimers

2.1. Organic Synthesis

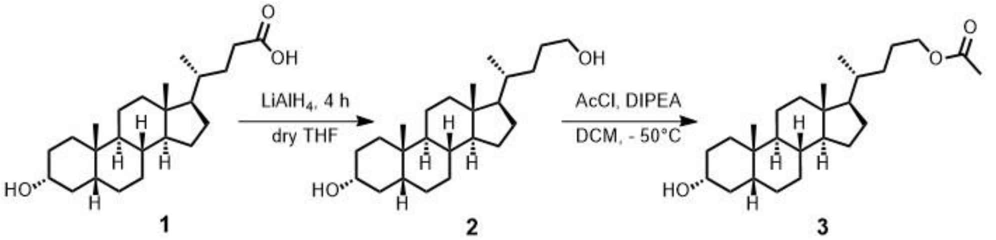

Figure S1. Synthesis pathway for compound **3.**

(3R,8R,9S,10S,13R,14S,17R)-17-((R)-5-hydroxypentan-2-yl)-10,13-dimethylhexadecahydro-1H-cyclopenta[a]phenanthren-3-ol (**2**) Synthesized according to published literature.[4]

$LiAlH_4$ (474 mg, 12.5 mmol, 4.7 eq.) was added slowly to a solution of lithocholic acid, **1** (1 g, 2.7 mmol, 1 eq.) in anhydrous THF (60 mL) at 0 °C. The reaction mixture was then stirred for 2.5 h at RT. H_2O (0.5 mL), 15 % aqueous NaOH (0.5 mL), and more H_2O (1.5 mL) were added sequentially to the reaction mixture. The white solid was removed by filtration. The organic layer was dried over Na_2SO_4, filtered, and concentrated under reduced pressure. The white solid was not further purified and was used as such in the next step (693 mg, 1.91 mmol, 72%). ^{1}H NMR **2** (300 MHz, $CDCl_3$) δ = 3.70 – 3.52 (m, 3H), 2.02 – 0.93 (m, 24H), 0.94 – 0.89 (m, 6H), 0.64 (s, 3H).

(4R)-4-((3R,8R,9S,10S,13R,14S,17R)-3-hydroxy-10,13-dimethylhexadecahydro-1H-cyclopenta[a]phenanthren-17-yl)pentyl acetate (**3**)

Acetyl chloride (23 µL, 0.33 mmol 1.2 eq.) dissolved in anhydrous THF (0.5 mL) was added dropwise under Ar to a mixture at -50 °C (dry ice, 1:1 ethanol/acetone) of **2** (99 mg, 0.27 mmol, 1 eq.) and DIPEA (0.1 mL, 0.59 mmol, 2.1 eq.) in anhydrous THF (4.5 mL). The reaction was stirred for 3 h at -50 °C, followed by 1 h at RT. The reaction mixture was then added to 2 mL of HCl (3.7%) and extracted with diethylether (3 x 10 mL). The organic phase was dried over Na_2SO_4, filtered, and concentrated under reduced pressure. The crude was purified by column chromatography (SiO_2, heptane/EtOAc 8:2; Rf=0.16). Compound **3** was isolated as a white solid (50 mg, 45%). ^{1}H NMR **3** (300 MHz, $CDCl_3$) δ = 4.01 (m, 2H), 3.62 (m, 1H), 2.04 (s, 3H), 2.00 – 0.94 (m, 21H), 0.91 (m, 6H), 0.64 (s, 3H). HRMS-NSI (*m/z*): $[M+K]^+$ calcd for $C_{26}H_{44}O_3K$, 443.2922; found, 433.2929.

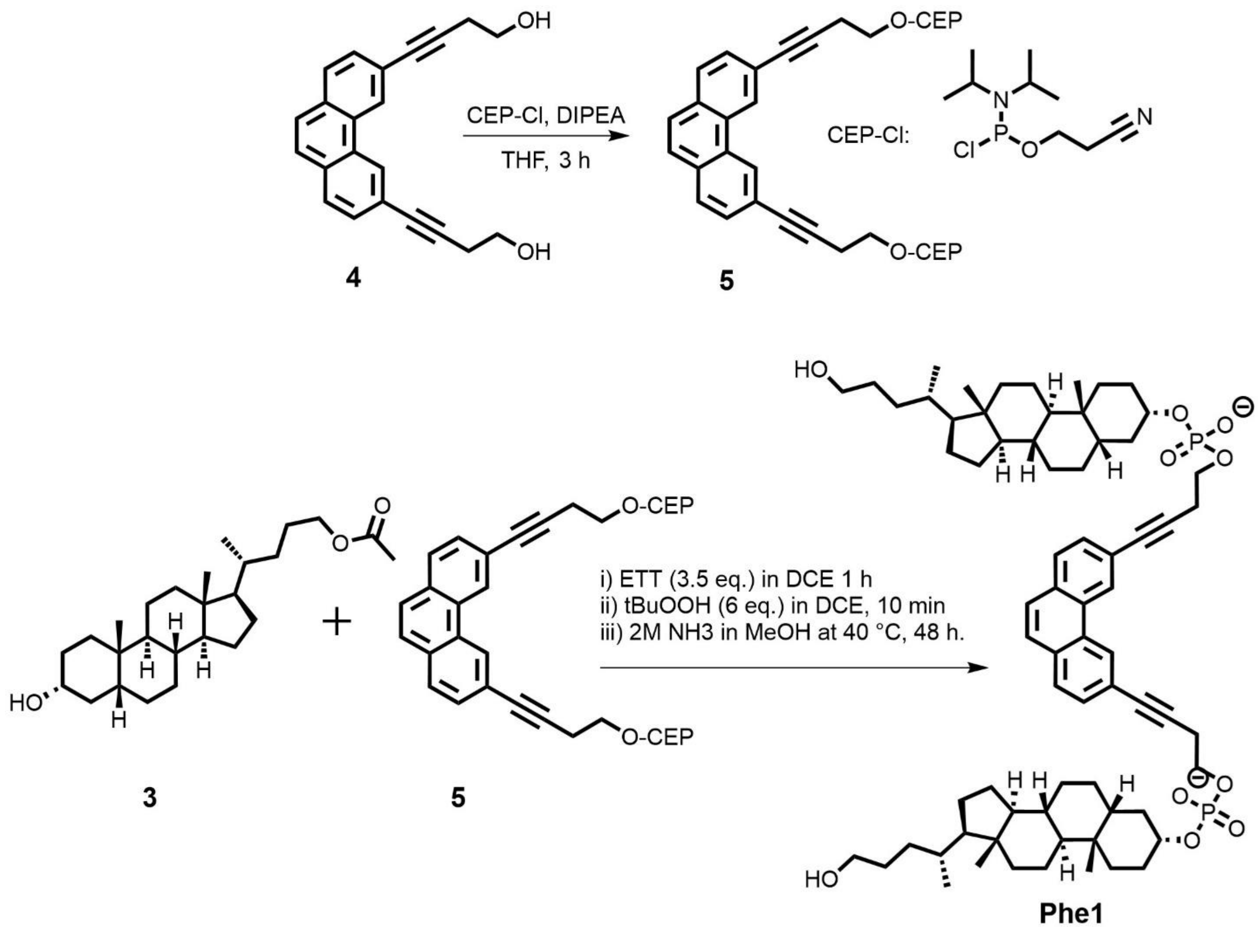

Figure S2 Synthesis pathway for **Phe1**.

bis(2-cyanoethyl)(phenanthrene-3,6-diylbis(but-3-yne-4,1-diyl))bis(diisopropylphosphoramidite) (**5**)

Compound **4** (50 mg, 0.2 mmol, 1 eq.) was dissolved in anhydrous THF (3 mL) and DIPEA (0.3 mL, 1.6 mmol, 10 eq.). 2-Cyanoethyl N,N-diisopropylchlorophosphoramidite (CEP-Cl, 78µL, 0.4 mmol, 2.2 eq.) was added dropwise at RT and the reaction mixture was stirred for 3 h under argon. The reaction mixture was concentrated under reduced pressure. The resultant yellow-greenish foam was purified by a short flash column chromatography (SiO_2, heptane/EtOAc 7:3 + 1% NEt_3). Compound **5** was isolated as a colorless oil (118 mg, 97%). ^{1}H NMR **5** (300 MHz, $CDCl_3$, sample contained traces of heptane) δ = 8.63 (d, J = 1.5 Hz, 2H), 7.72 (d, J = 8.2 Hz, 2H), 7.61 (s, 2H), 7.52 (dd, J = 8.2 Hz, 1.4 Hz, 2H), 3.82 (m, 7H), 3.59 (m, 3H), 2.75 (t, J = 7.0 Hz, 4H), 2.60 (m, 4H), 1.16 (dd, J = 6.8 Hz, 5.7 Hz, 29H). ^{31}P NMR **5** (121 MHz, $CDCl_3$) δ = 148.22. HRMS-NSI (m/z): $[M+H]^+$ calcd for $C_{40}H_{53}N_4O_4P_2$, 715.3537; found, 715.3546.

Trimer **Phe1**

To a solution of **3** (32 mg, 0.1 mmol, 3 eq.) and **5** (20 mg, 0.03 mmol, 1 eq.) in 1,2 dichloroethane (DCE, 0.95 mL) was added a solution of 5-(ethylthio)-1H-tetrazole (12 mg, 0.1 mmol, 3.5 eq.) in DCE (0.31 ml) under argon atmosphere. The reaction was stirred at RT for 3 h after which a solution of ^{t}BuOOH (70% in water, 23 µL, 0.2 mmol) was added. After 10 minutes, the reaction mixture was diluted with $CHCl_3$ (15 mL) and washed with aq. sat. $NaHCO_3$ (15 mL) and brine (15 mL). The organic layer was dried over $MgSO_4$, filtrated and concentrated under reduced pressure. The crude was purified by preparative TLC

(DCM/toluene/MeOH 86:10:4). The protected trimer was isolated as a brown solid. This latter was further deprotected with 2M NH_3 in MeOH (5 mL) for 72 h at 40 °C and purified by RP-HPLC. HRMS-NSI (m/z): $[M-H]^-$ calcd for $C_{70}H_{98}O_{10}P_2$, 580.3323; found, 580.3310.

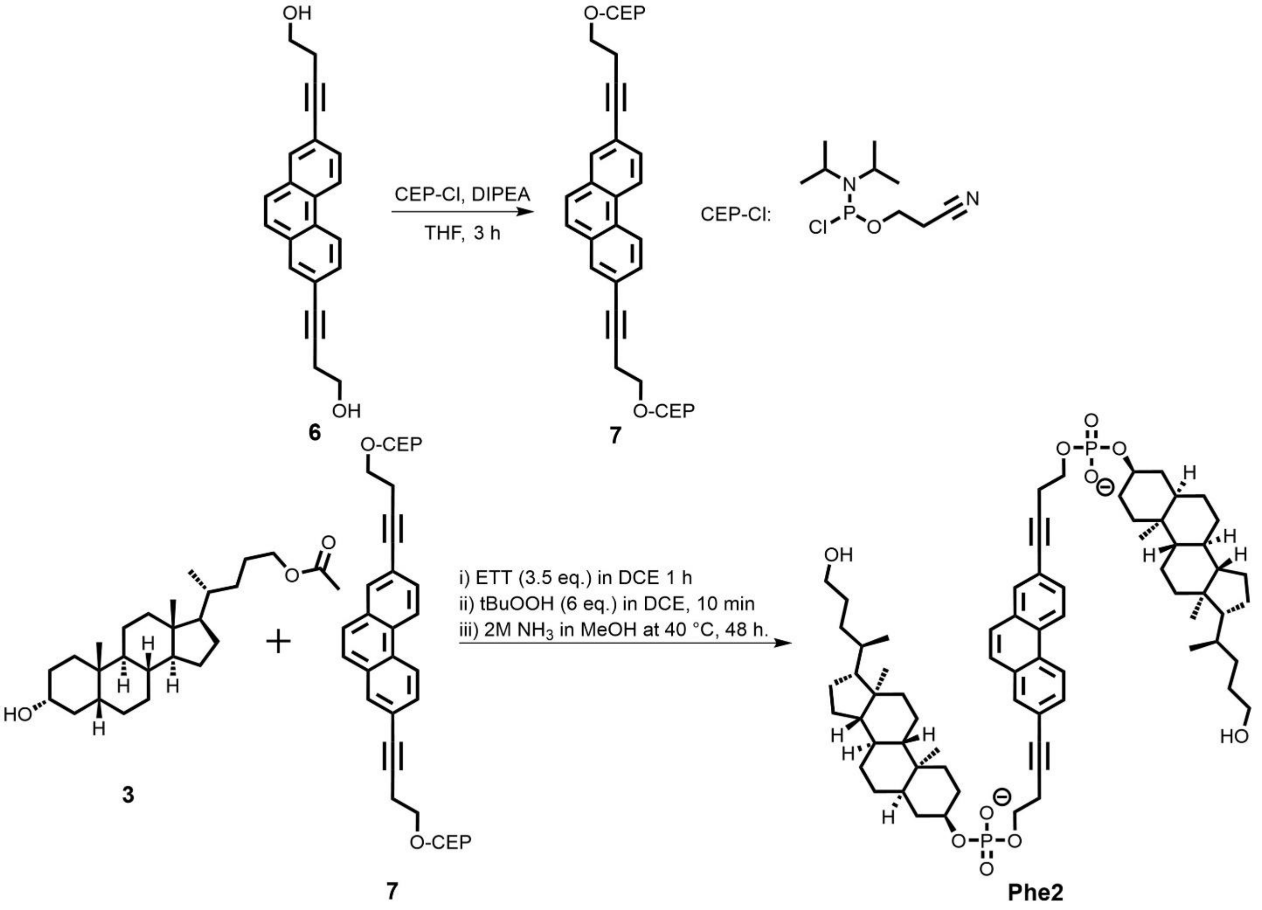

Figure S3. Synthesis pathway for **Phe2**.

bis(2-cyanoethyl)(phenanthrene-2,7-diylbis(but-3-yne-4,1-diyl))bis(diisopropylphosphoramidite) (**6**)

Compound **6** (50 mg, 0.2 mmol, 1 eq.) was dissolved in anhydrous THF (3 mL) and DIPEA (0.3 mL, 1.48 mmol, 10 eq.). 2-Cyanoethyl N,N-diisopropylchlorophosphoramidite (CEP-Cl, 78µL, 0.4 mmol, 2.2 eq.) was added dropwise at RT and the reaction mixture was stirred for 3 h under argon. The reaction mixture was concentrated under reduced pressure. The resultant yellow-greenish foam was purified by a short flash column chromatography (SiO_2, heptane / EtOAc / NEt_3 7:3:1%). Compound **7** was isolated as a colorless oil (104 mg, 91%). ^1H NMR **7** (300 MHz, $CDCl_3$; sample contained traces of heptane) δ = 8.53 (d, J = 8.7 Hz, 2H), 7.92 (d, J = 1.7 Hz, 2H), 7.69 – 7.59 (m, 4H), 4.01 – 3.74 (m, 8H), 3.65 (m, 4H), 2.80 (t, J = 6.9 Hz, 4H), 2.66 (m, 4H), 1.28 – 1.17 (m, 29H). ^{31}P NMR **7** (121 MHz, $CDCl_3$) δ = 148.21. HRMS-NSI (m/z): [M+H]+ calcd for $C_{40}H_{53}N_4O_4P_2$, 715.3537; found, 715.3552.

Trimer **Phe2**

To a solution of **7** (20 mg, 0.03 mmol, 1 eq.) in DCE (0.20 mL) was added 5-(ethylthio)-1H-tetrazole (13 mg, 0.1 mmol, 3.5 eq.) in DCE (0.32 mL) under argon atmosphere. Compound **3** (34 mg, 0.1 mmol, 3 eq.) was

dissolved in DCE (0.66 mL) and added to the activated compound **7**. The reaction was stirred at RT for 3 h. Tert-butyl hydroperoxide solution (70% in water, 23 µL, 0.2 mmol) was added. After 10 minutes, the reaction was diluted with $CHCl_3$ (15 mL) and washed with aq. sat. $NaHCO_3$ (15 mL) and brine (15 mL). The organic layer was dried over Na_2SO_4, filtered, and concentrated under reduced pressure. The crude was purified by a preparative TLC (DCM / toluene / MeOH 86:10:4). The protected trimer was isolated as a brown solid. This latter was further deprotected with 2M NH_3 in MeOH (6 mL) for 48 h at 40 °C and purified by RP-HPLC. HRMS-NSI (*m/z*): $[M-H]^-$ calcd for $C_{70}H_{98}O_{10}P_2$, 580.3323; found, 580.3312.

Trimer Purification

After the deprotection, the trimer was purified by reverse-phase HPLC (Shimadzu LC-20AT, ReproSil 100 C8, 5,0 µm, 250×4 mm) at 40 °C with a flow rate of 1 mL/min. Solvent A: aqueous 2.1 mM triethylamine (TEA) / 25 mM 1,1,1,3,3,3-hexafluoropropan-2-ol (HFIP) pH 8; solvent B: acetonitrile; gradient: B [%] t_R [min]: 40 (0), 40 (2), 60 (24). The purified cholane-phenanthrene-cholane trimers **Phe1** and **Phe2** were dissolved in 1 mL of 80% EtOH in Milli-Q H_2O. The absorbance was measured to determine the concentration of the stock solutions of **Phe1** and **Phe2**. The Beer-Lambert law was applied to determine the concentration with the molar absorption coefficients in $L \cdot mol^{-1} \cdot cm^{-1}$ were used: ε(3,6-Phe, 326 nm): 35’400; ε(2,7-Phe, 260 nm): 47’000.

2.2. NMR Spectra

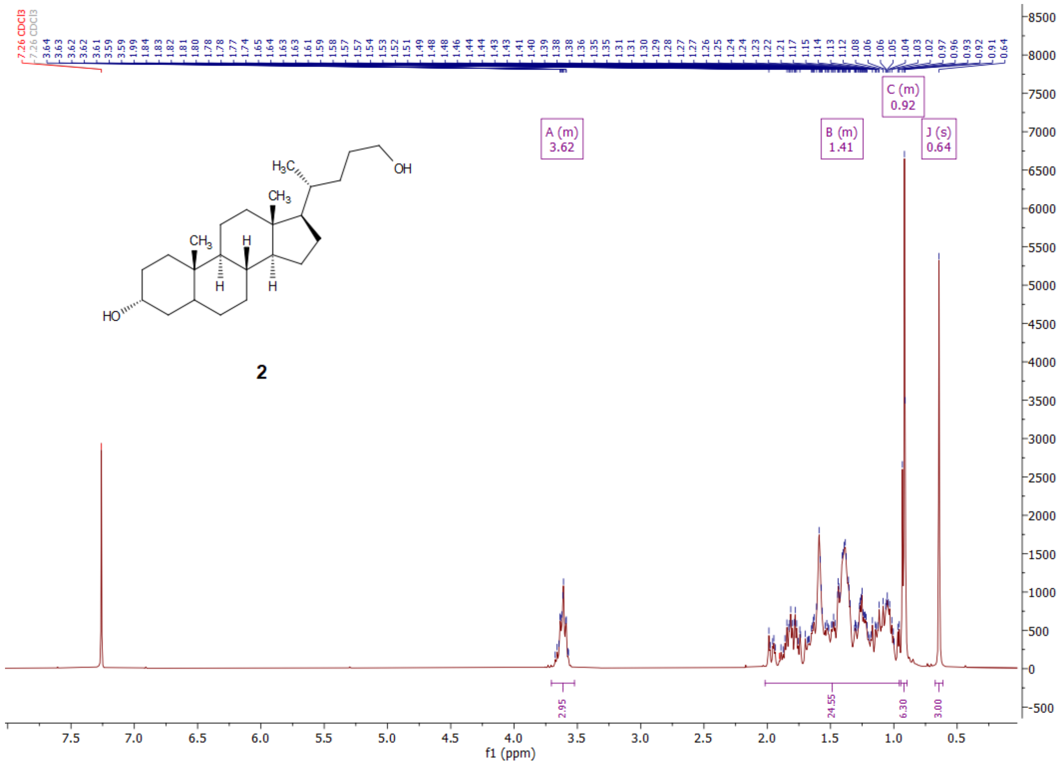

Figure S4 ^{1}H NMR spectra of **2**.

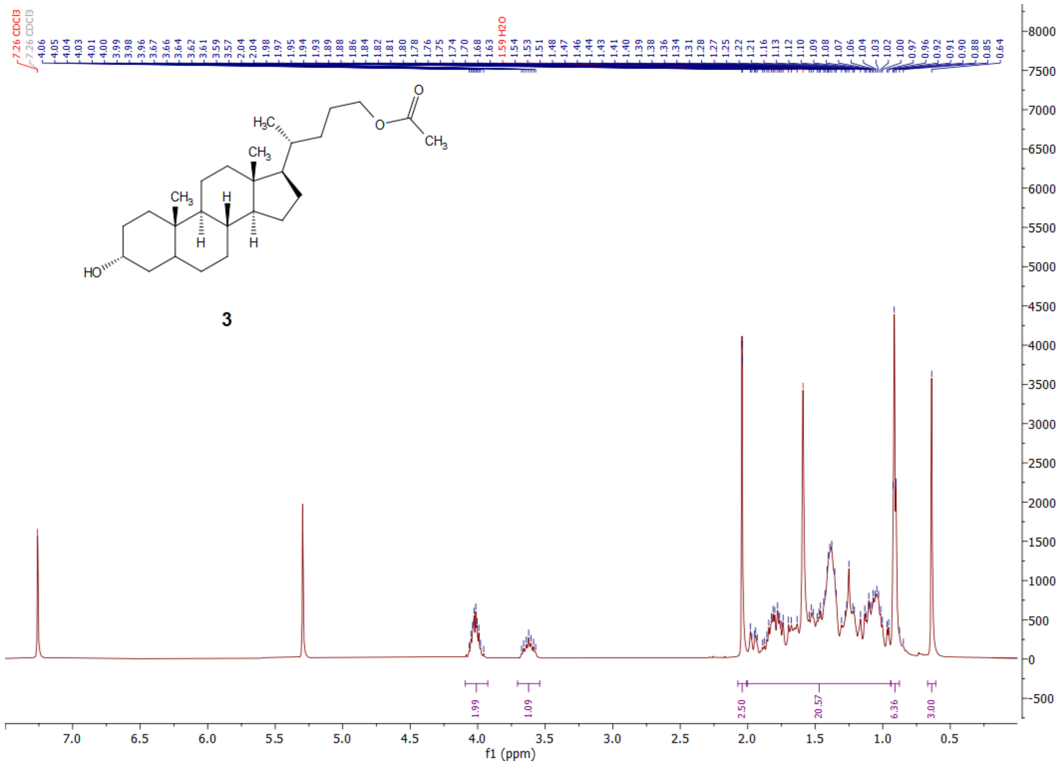

Figure S5 ^{1}H NMR spectra of **3**.

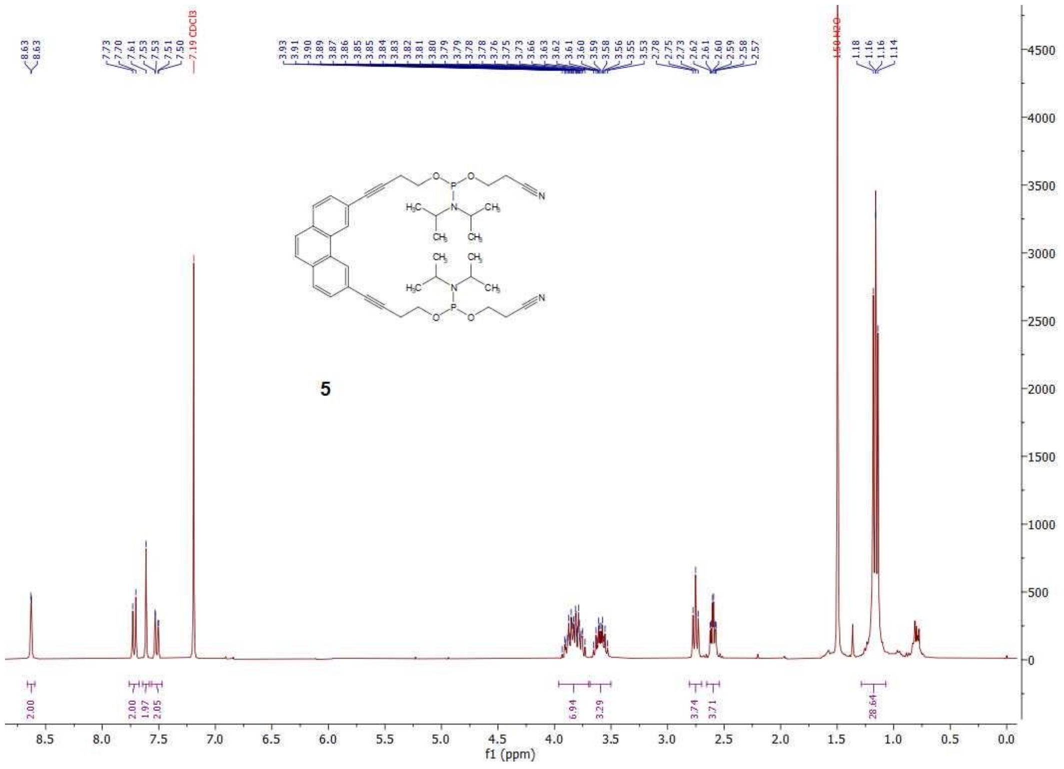

Figure S6 ^{1}H NMR spectra of **5**.

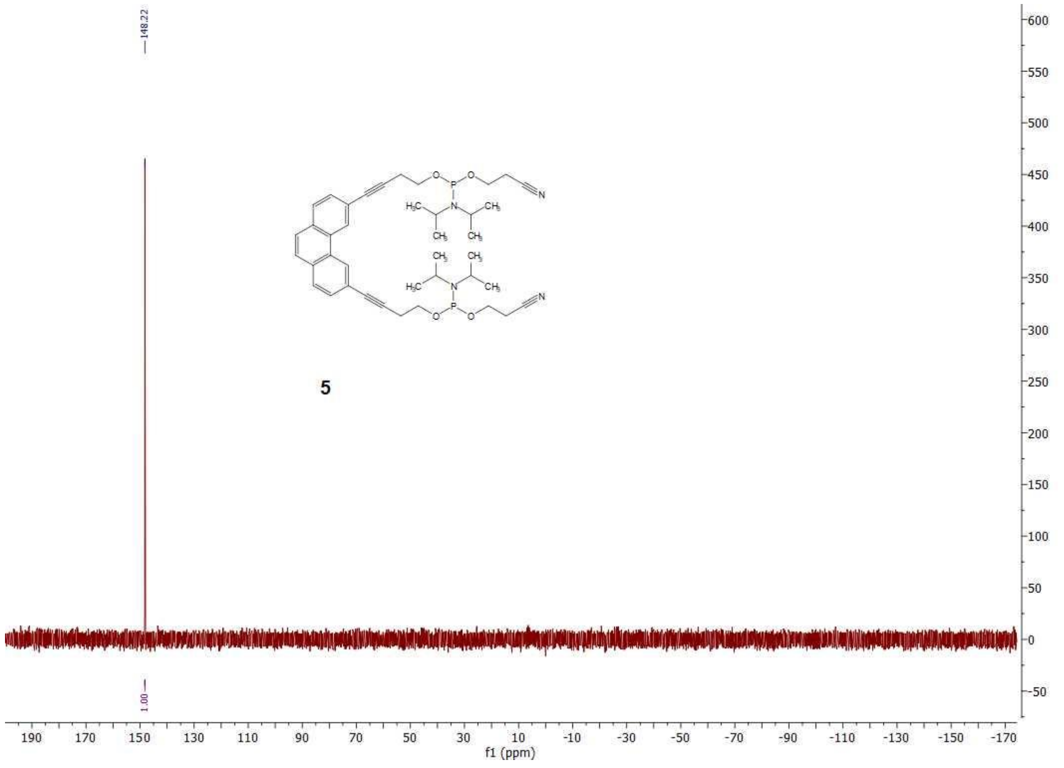

Figure S7 ^{31}P NMR spectra of **5**.

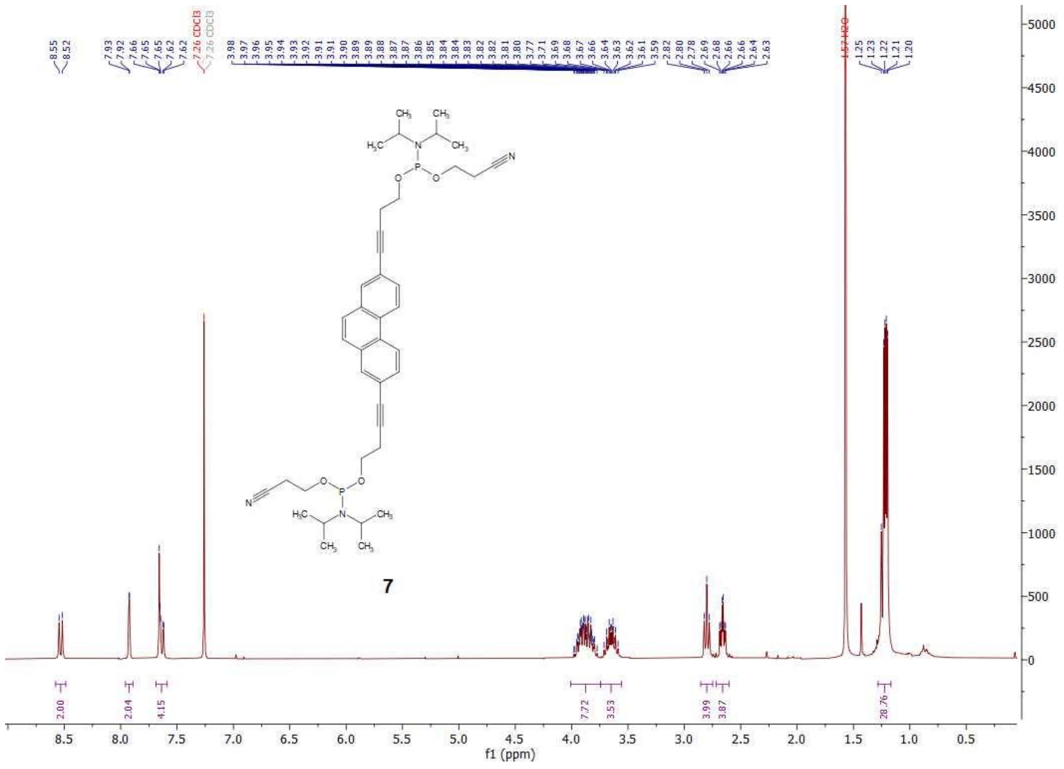

Figure S8. ^{1}H NMR spectra of **7**.

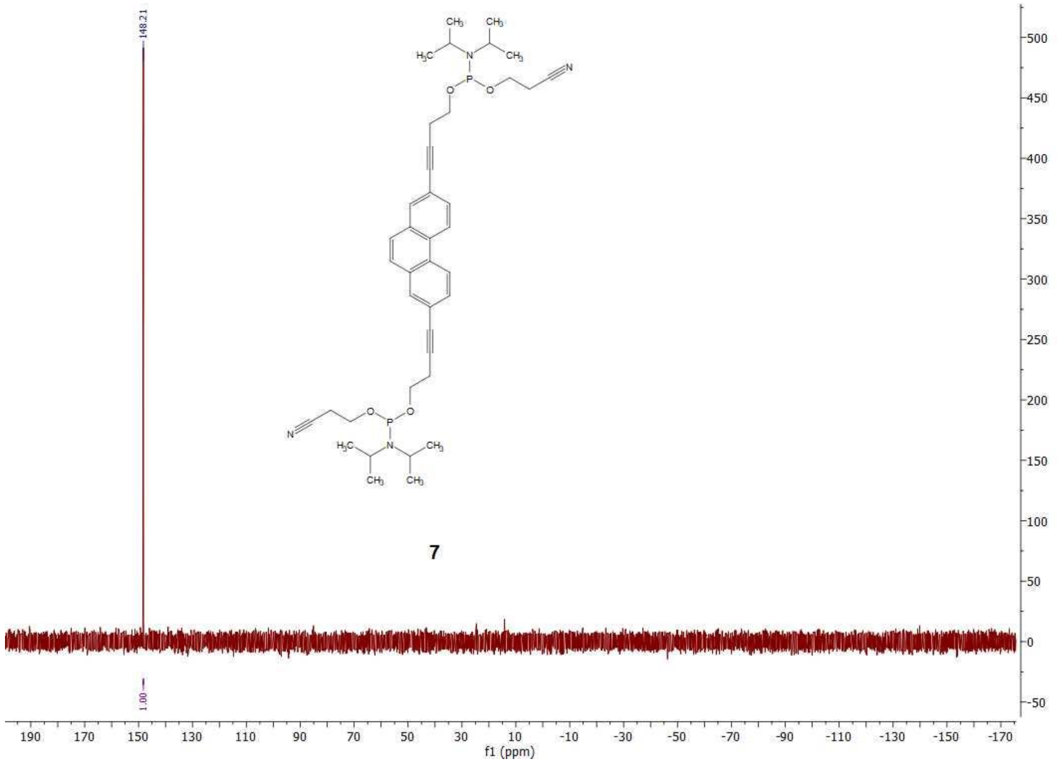

Figure S9. ^{31}P NMR spectra of **7**.

2.3. MS Spectra

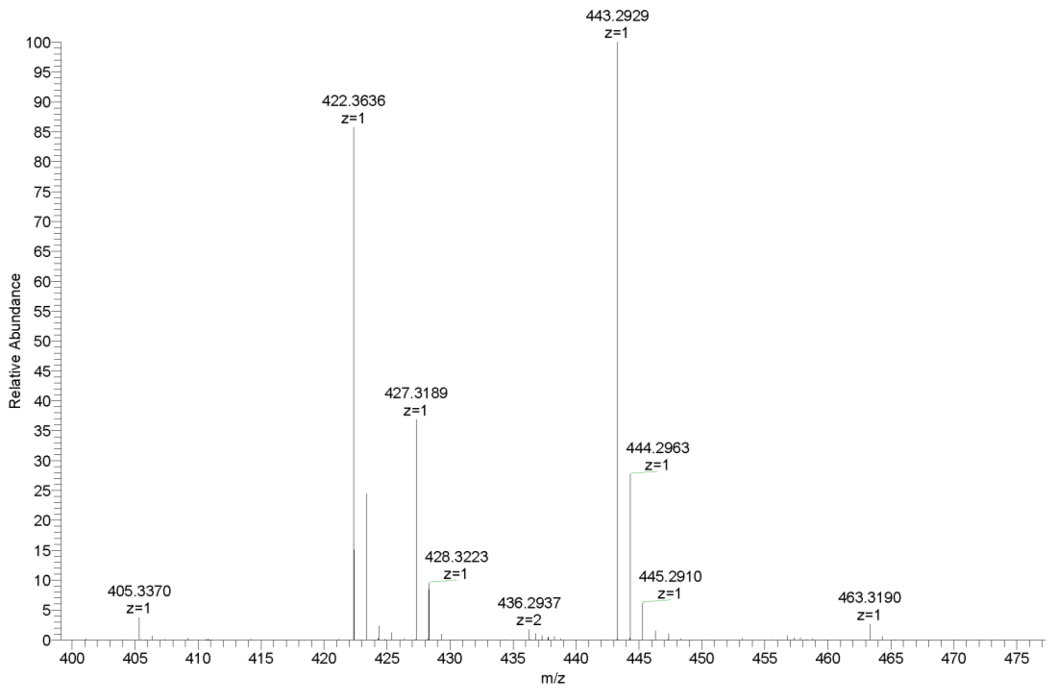

Figure S10 HR-MS for **3** in presence of a K^+ adduct.

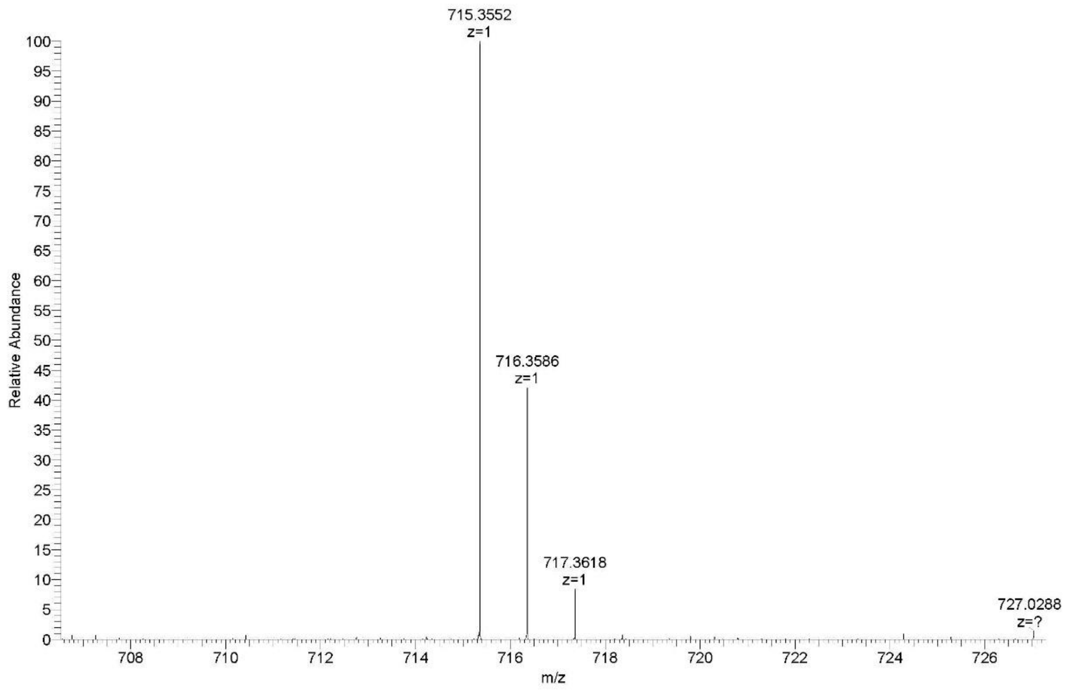

Figure S11 HR-MS for compound **5**.

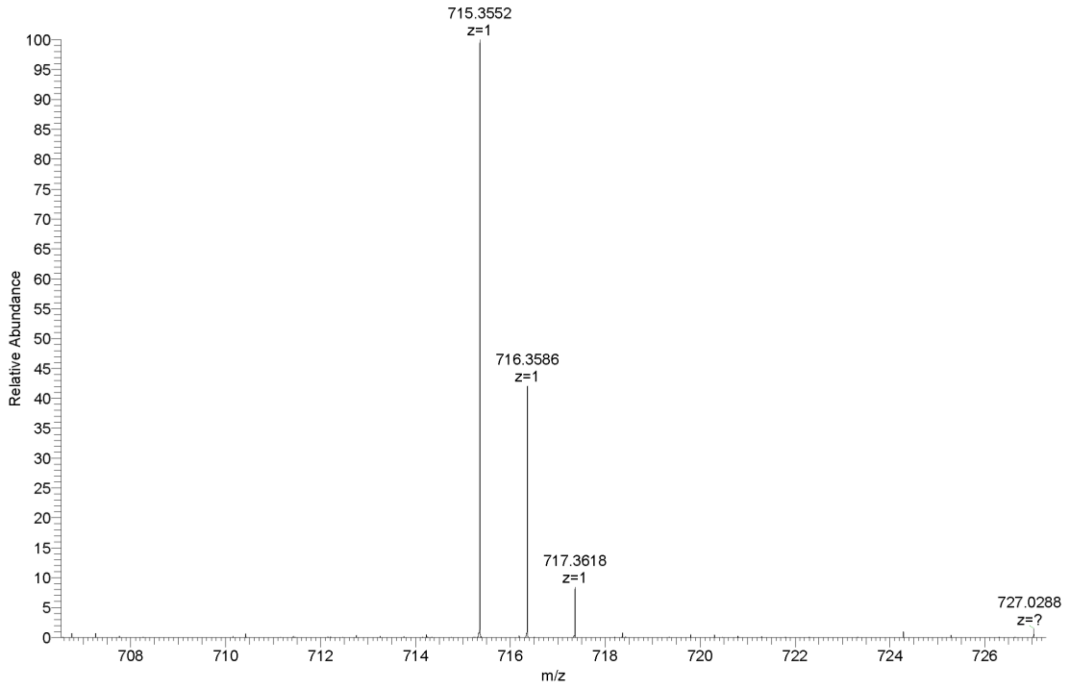

Figure S12 HR-MS for compound **7**.

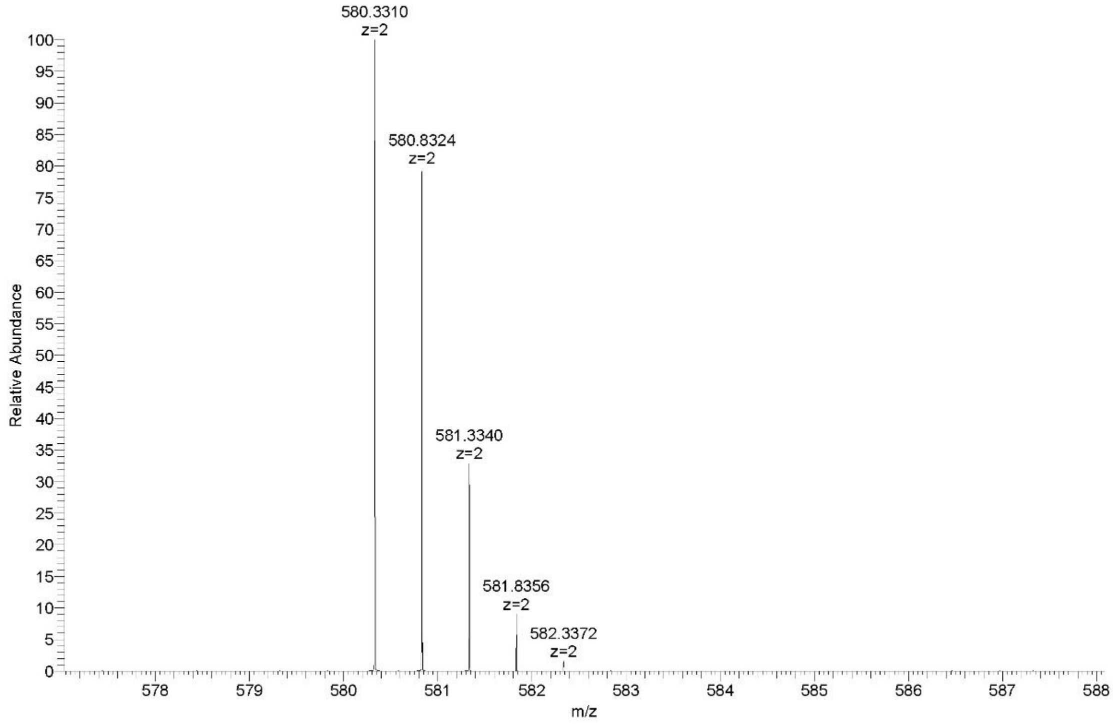

Figure S13 HR-MS of trimer **Phe1**.

Elemental composition search on mass 580.3310

m/z= 575.3310-585.3310

m/z	Theo. Mass	Delta (ppm)	Composition
580.3310	580.3306	0.83	$C_{84}H_{89}O_2P$
	580.3323	-2.19	$C_{70}H_{98}O_{10}P_2$
	580.3294	2.87	$C_{77}H_{94}O_5P_2$
	580.3335	-4.23	$C_{77}H_{93}O_7P$

Figure S14 Elemental composition search for **Phe1**.

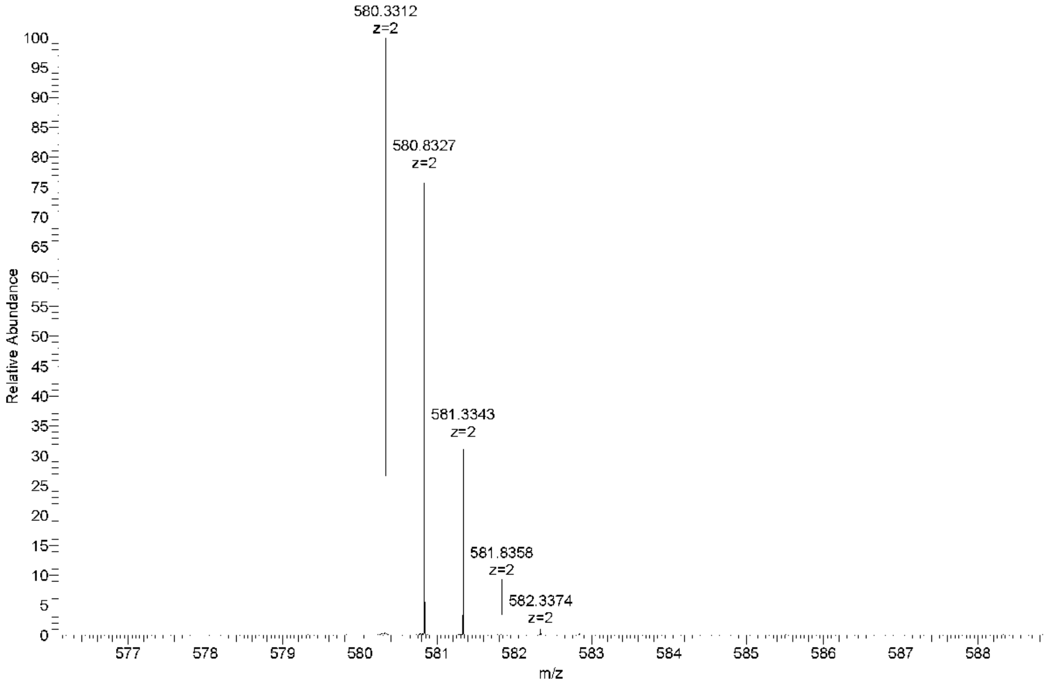

Figure S15 HR-MS of trimer **Phe2**.

Elemental composition search on mass 580.3312

m/z= 575.3312-585.3312

m/z	Theo. Mass	Delta (ppm)	Composition
580.3312	580.3311	0.13	$C_{68}H_{99}O_{10}NaP_2$
	580.3313	-0.25	$C_{76}H_{90}O_9N$
	580.3307	0.80	$C_{80}H_{92}O_2NP_2$
	580.3306	1.07	$C_{84}H_{89}O_2P$
	580.3301	1.79	$C_{69}H_{95}O_{12}NP$
	580.3301	1.82	$C_{74}H_{91}O_9NNa$
	580.3323	-1.92	$C_{75}H_{94}O_7NaP$
	580.3323	-1.95	$C_{70}H_{98}O_{10}P_2$
	580.3300	2.06	$C_{73}H_{92}O_{12}$
	580.3324	-2.18	$C_{71}H_{97}O_7NNaP_2$

Figure S16 Elemental composition search for **Phe2**.

2.4. HPLC Trace

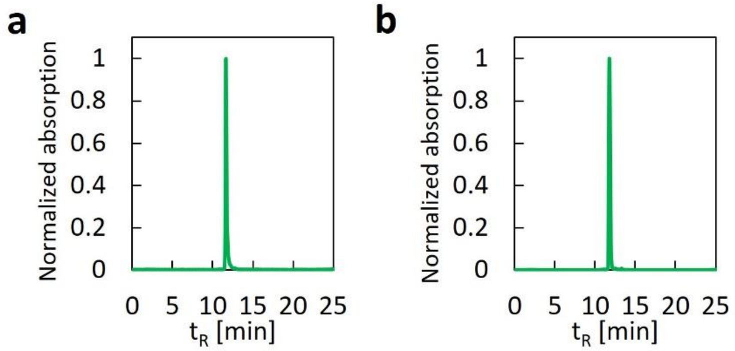

Figure S17 HPLC traces of(a) **Phe1**, $t_{[R]}$: 11.7 min; (b) **Phe2,** $t_{[R]}$: 11.8 min.

3. Preparation of the samples

In an Eppendorf was added subsequently Milli-Q H_2O, EtOH, 100 µL of a sodium phosphate buffer (0.1 M, pH 7.2), 5 µL of NaCl (2 M) and finally the trimer for a final volume of 1 mL. The solution was then vortexed to ensure a thorough mixing before being transferred to a quartz cuvette.

For the doping experiments, a small amount (0.5 µL to 2 µL) of the acceptor trimer was added to the previous sample. The solution was then heated to ensure a full dissociation of the aggregates and cooled down following a temperature gradient (0.5 °C/min or 10 °C /min).

4. Temperature-dependent UV-vis Spectra

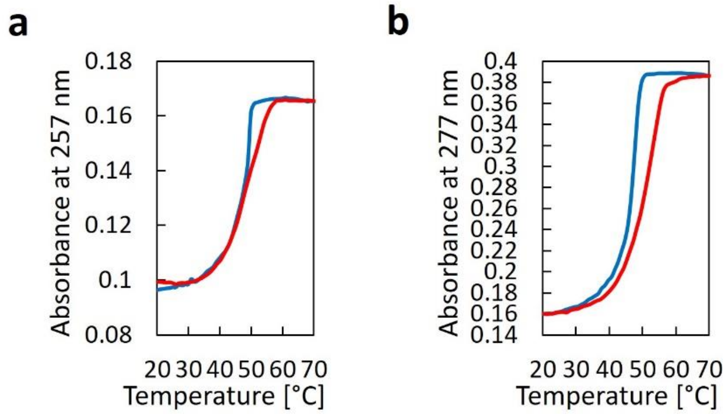

Figure S18 UV-vis absorbance monitored at one specific wavelength over cooling from 70 °C to 20 °C (blue) and heating back to 70 °C (red) of **Phe1** $\lambda_{abs.}$: 257 nm (gradient 0.5 °C·min^{-1}), **Phe2** $\lambda_{abs.}$: 277 nm (gradient 0.5 °C·min^{-1}). Conditions: 3 µM trimer, 10 mM sodium phosphate buffer, 10 mM NaCl and EtOH (20% EtOH for **Phe1**, 15% EtOH for **Phe2**).

5. Atomic Force Microscopy

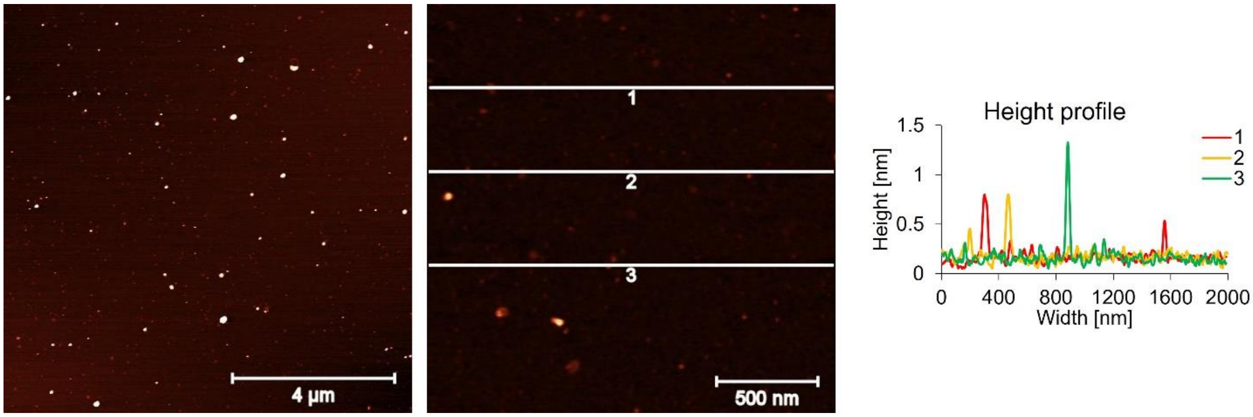

Figure S19 AFM images of the buffer used for **Phe1** on an APTES-modified mica sheet with cross-section (right). Conditions: 10 mM sodium phosphate buffer pH 7.2, 10 mM NaCl and 15% EtOH

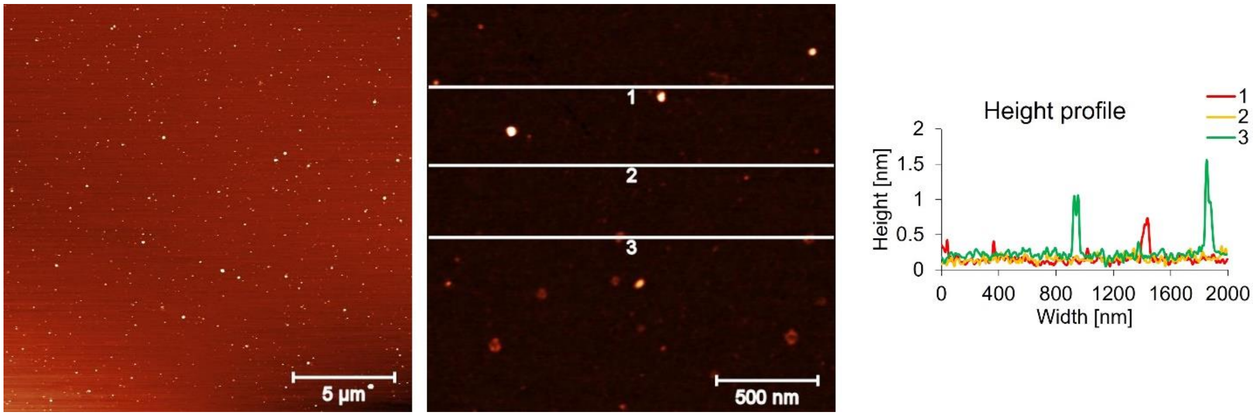

Figure S20 AFM images of the buffer used for **Phe2** on an APTES-modified mica sheet with cross-section (right). Conditions: 10 mM sodium phosphate buffer pH 7.2, 10 mM NaCl and 20% EtOH.

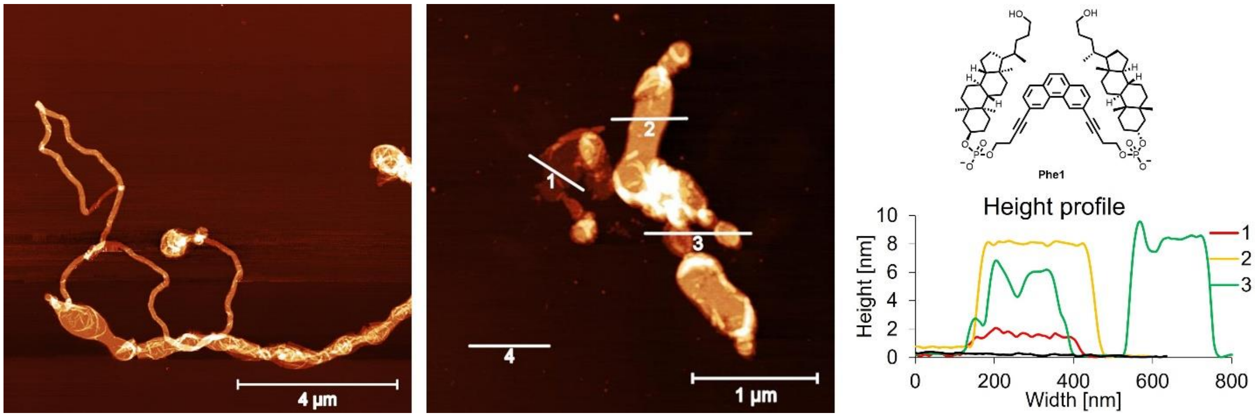

Figure S21 AFM images of the self-assembled **Phe1** (temperature gradient: 0.5°C per min) on an APTES-modified mica with cross-section (right). Conditions: 3 µM **Phe1**, 10 mM sodium phosphate buffer pH 7.2, 10 mM NaCl and 15% EtOH.

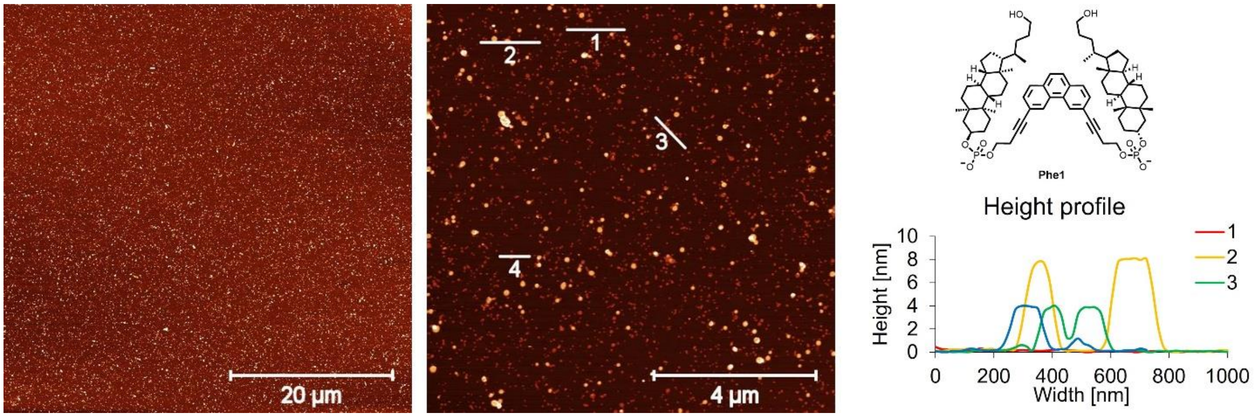

Figure S22 AFM images of the self-assembled **Phe1** (temperature gradient: 10 °C per min) on an APTES-modified mica with cross-section (right). Conditions: 3 µM **Phe1**, 10 mM sodium phosphate buffer pH 7.2, 10 mM NaCl and 15% EtOH.

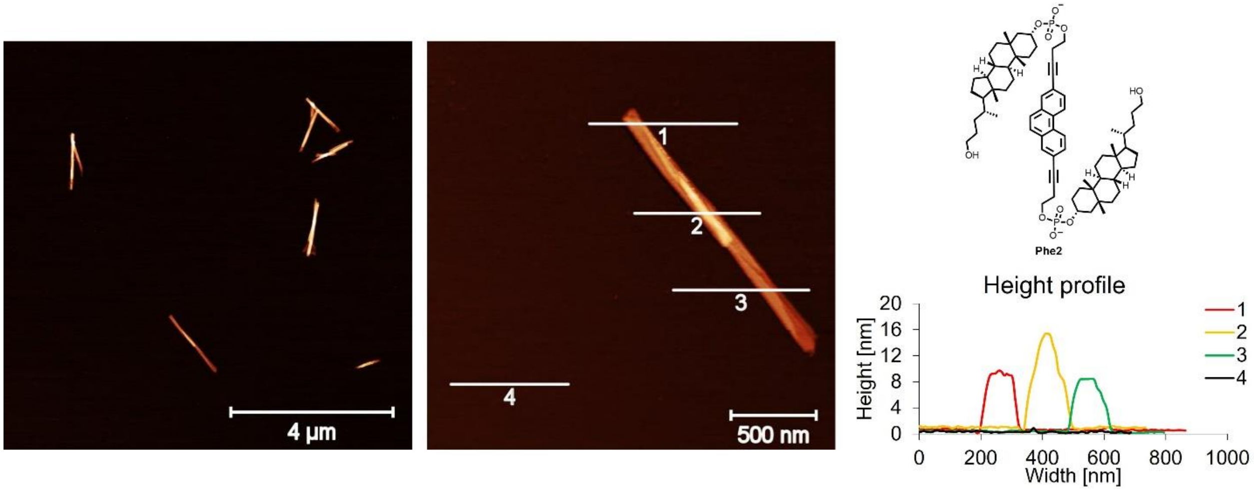

Figure S23 AFM images of the self-assembled **Phe2** (temperature gradient: 0.5°C per min) on an APTES-modified mica with cross-section (right). Conditions: 3 µM **Phe2**, 10 mM sodium phosphate buffer pH 7.2, 10 mM NaCl and 20% EtOH.

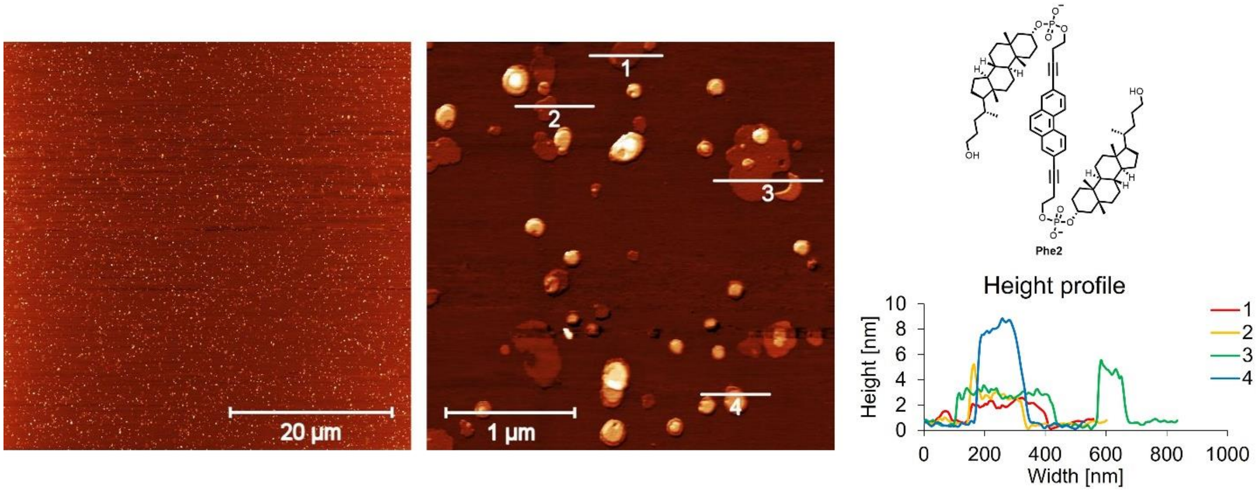

Figure S24 AFM images of the self-assembled **Phe2** (temperature gradient: 10 °C per min) on an APTES-modified mica with cross-section (right). Conditions: 3 µM **Phe2**, 10 mM sodium phosphate buffer pH 7.2, 10 mM NaCl and 20% EtOH.

6. Cryo-EM

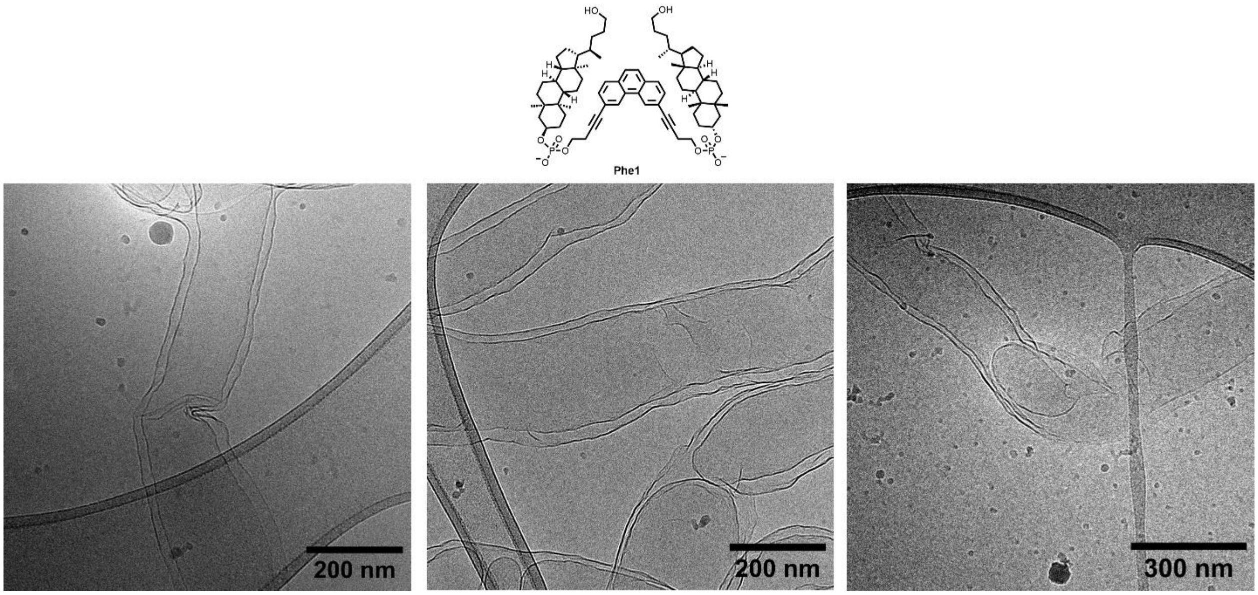

Figure S25 Cryo-EM images of **Phe1**. Conditions: 3 µM **Phe1**, 10 mM sodium phosphate buffer pH 7.2, 10 mM NaCl and 15% EtOH.

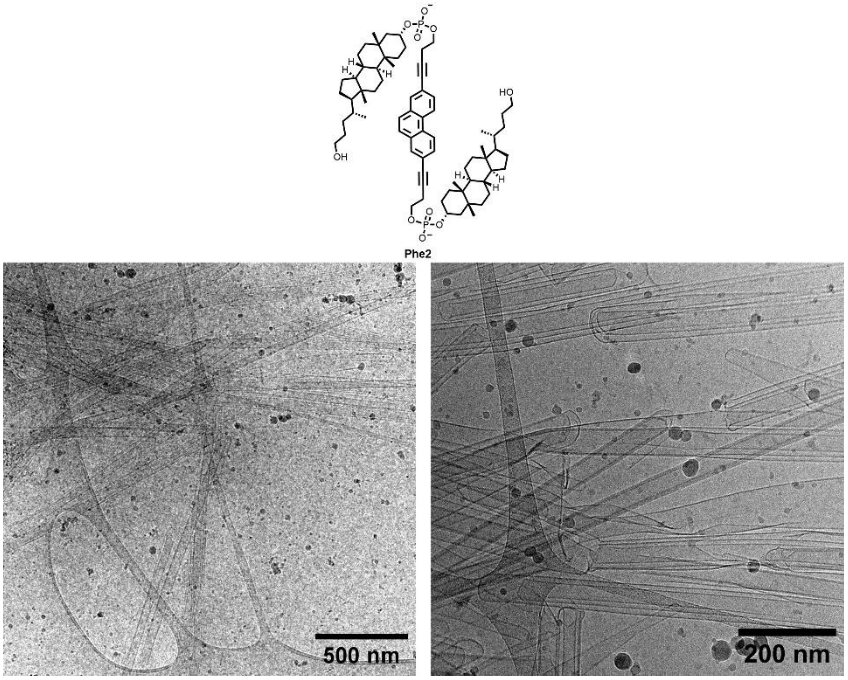

Figure S26 Cryo-EM images of **Phe2**. Conditions: 3 µM **Phe2**, 10 mM sodium phosphate buffer pH 7.2, 10 mM NaCl and 20% EtOH.

7. Quantum Yield Calculations

Table S1. Quantum yield calculation table for **Phe1** with increasing amounts of **Py1**. Quinine sulfate in 0.5 M H_2SO_4 was used as a reference – Φ_R^{QS} = 0.546. Conditions: 0.5 °C/min cooling gradient, 3 µM **Phe1**, 10 mM sodium phosphate buffer pH 7.2, 10 mM NaCl and 15% EtOH.

	I_S	I_R	$A_{S - 333\ nm}$	$A_{R - 333\ nm}$	ϕ_S	ϕ_F
	3891	41780	0.083	0.088	5.4	
0.0%	3887	42737	0.083	0.093	5.5	5.5 ± 0.2
	3788	42523	0.085	0.100	5.7	
	9277	41780	0.079	0.088	13.4	
0.3%	9014	42737	0.081	0.093	13.2	13.7 ± 0.7
	9530	42523	0.085	0.100	14.5	
	12513	41780	0.080	0.088	17.9	
0.6%	12902	42737	0.081	0.093	18.8	19.1 ± 1.4
	13390	42523	0.084	0.100	20.6	
	20193	41780	0.079	0.088	29.2	
1.5%	20514	42737	0.081	0.093	30.0	30.2 ± 1.2
	20598	42523	0.084	0.100	31.5	
	26167	41780	0.082	0.088	36.7	
3.0%	27586	42737	0.082	0.093	39.6	39.4 ± 2.6
	27676	42523	0.085	0.100	41.9	
	29653	41780	0.088	0.088	38.4	
6.0%	30396	42737	0.092	0.093	39.1	40.9 ± 3.7
	30868	42523	0.088	0.100	45.1	
	27734	41780	0.082	0.088	38.5	
12.0%	28448	42737	0.083	0.093	40.6	40.6 ± 2.0
	29260	42523	0.089	0.100	42.6	
	22899	41780	0.083	0.088	31.7	
24.0%	23302	42737	0.084	0.093	32.7	33.0 ± 1.5
	24235	42523	0.090	0.100	34.7	

Table S2. Quantum yield calculation table for **Phe1** with increasing amounts of **Py1**. Quinine sulfate in 0.5 M H_2SO_4 was used as a reference – Φ_R^{QS} = 0.546. Conditions: 10 °C/min cooling gradient, 3 µM **Phe1**, 10 mM sodium phosphate buffer pH 7.2, 10 mM NaCl and 15% EtOH.

	I_S	I_R	$A_{S - 333\ nm}$	$A_{R - 333\ nm}$	ϕ_S	ϕ_F
0.0%	4854	41780	0.080	0.088	7.0	7.3 ± 0.4
	4915	42737	0.082	0.093	7.1	
	4975	42523	0.082	0.100	7.8	
0.3%	9456	41780	0.077	0.088	14.1	14.9 ± 1.4
	9471	42737	0.079	0.093	14.1	
	10149	42523	0.079	0.100	16.5	
0.6%	12774	41780	0.078	0.088	18.8	19.9 ± 1.2
	13228	42737	0.079	0.093	19.8	
	13528	42523	0.082	0.100	21.2	
1.5%	20320	41780	0.078	0.088	29.9	31.3 ± 1.6
	20409	42737	0.078	0.093	31.0	
	20448	42523	0.080	0.100	33.0	
3.0%	26298	41780	0.079	0.088	38.2	41.2 ± 3.0
	27569	42737	0.079	0.093	41.3	
	27752	42523	0.081	0.100	44.2	
6.0%	29695	41780	0.084	0.088	40.5	41.9 ± 2.6
	30382	42737	0.089	0.093	40.4	
	30848	42523	0.089	0.100	44.9	
12.0%	28129	41780	0.085	0.088	37.9	39.6 ± 2.3
	28594	42737	0.087	0.093	38.9	
	29477	42523	0.090	0.100	42.2	
24.0%	24146	41780	0.081	0.088	34.1	35.7 ± 1.7
	24632	42737	0.082	0.093	35.4	
	25355	42523	0.087	0.100	37.4	

Table S3. Quantum yield calculation table for **Phe1** with increasing amounts of **Py2**. Quinine sulfate in 0.5 M H_2SO_4 was used as a reference – Φ_R^{QS} = 0.546. Conditions: 0.5 °C/min cooling gradient, 3 µM **Phe1**, 10 mM sodium phosphate buffer pH 7.2, 10 mM NaCl and 15% EtOH.

	I_S	I_R	$A_{S - 333\ nm}$	$A_{R - 333\ nm}$	ϕ_S	ϕ_F
	4485	41780	0.067	0.088	7.6	
0.0%	4594	42737	0.064	0.093	8.5	8.2 ± 0.5
	4755	42523	0.073	0.100	8.4	
	6800	41780	0.067	0.088	11.6	
0.3%	6883	42737	0.063	0.093	12.8	12.2 ± 0.6
	6907	42523	0.073	0.100	12.1	
	8477	41780	0.067	0.088	14.4	
0.6%	8649	42737	0.065	0.093	15.8	14.4 ± 1.3
	8579	42523	0.084	0.100	13.1	
	13795	41780	0.068	0.088	23.4	
1.5%	13926	42737	0.066	0.093	25.1	23.9 ± 1.0
	14191	42523	0.078	0.100	23.3	
	19778	41780	0.069	0.088	32.7	
3.0%	20777	42737	0.067	0.093	36.5	35.0 ± 2.0
	21541	42523	0.078	0.100	35.8	
	22530	41780	0.070	0.088	36.8	
6.0%	24006	42737	0.067	0.093	42.2	40.4 ± 3.2
	25324	42523	0.077	0.100	42.3	
	18287	41780	0.072	0.088	28.9	
12.0%	20426	42737	0.069	0.093	35.0	33.6 ± 4.1
	22479	42523	0.079	0.100	36.8	
	13787	41780	0.074	0.088	21.3	
24.0%	14455	42737	0.072	0.093	23.6	23.2 ± 1.7
	15455	42523	0.081	0.100	24.6	

Table S4. Quantum yield calculation table for **Phe1** with increasing amounts of **Py2**. Quinine sulfate in 0.5 M H_2SO_4 was used as a reference – Φ_R^{QS} = 0.546. Conditions: 10 °C/min cooling gradient, 3 µM **Phe1**, 10 mM sodium phosphate buffer pH 7.2, 10 mM NaCl and 15% EtOH.

	I_S	I_R	$A_{S - 333 nm}$	$A_{R - 333 nm}$	ϕ_S	ϕ_F
0.0%	4496	41780	0.068	0.088	7.6	8.3 ± 0.6
	4770	42737	0.064	0.093	8.8	
	4804	42523	0.073	0.100	8.5	
0.3%	7241	41780	0.065	0.088	12.7	13.6 ± 0.9
	7373	42737	0.060	0.093	14.4	
	7453	42523	0.070	0.100	13.8	
0.6%	10068	41780	0.066	0.088	17.5	17.8 ± 1.8
	10442	42737	0.062	0.093	19.8	
	10263	42523	0.082	0.100	16.2	
1.5%	15023	41780	0.066	0.088	25.9	27.2 ± 1.7
	15487	42737	0.063	0.093	29.1	
	15630	42523	0.076	0.100	26.5	
3.0%	19732	41780	0.067	0.088	33.9	36.9 ± 2.7
	20961	42737	0.065	0.093	38.4	
	21775	42523	0.073	0.100	38.6	
6.0%	22149	41780	0.068	0.088	37.1	40.8 ± 3.2
	23504	42737	0.065	0.093	42.6	
	24991	42523	0.075	0.100	42.8	
12.0%	21735	41780	0.070	0.088	35.4	39.5 ± 3.7
	22891	42737	0.067	0.093	40.4	
	24952	42523	0.075	0.100	42.7	
24.0%	16797	41780	0.072	0.088	26.7	28.8 ± 2.4
	17456	42737	0.073	0.093	28.3	
	19036	42523	0.078	0.100	31.4	

Table S5. Quantum yield calculation table for **Phe2** with increasing amounts of **Py2**. Quinine sulfate in 0.5 M H_2SO_4 was used as a reference – Φ_R^{QS} = 0.546. Conditions: 0.5 °C/min cooling gradient, 3 µM **Phe2**, 10 mM sodium phosphate buffer pH 7.2, 10 mM NaCl and 20% EtOH.

	I_S	I_R	$A_{S - 320\ nm}$	$A_{R - 320\ nm}$	ϕ_S	ϕ_F
	7433	41780	0.089	0.083	9.0	
0.0%	6886	42737	0.077	0.088	10.0	10.0 ± 1.0
	6713	42523	0.074	0.095	11.1	
	12582	41780	0.089	0.083	15.3	
0.3%	12094	42737	0.077	0.088	17.5	17.6 ± 2.3
	12119	42523	0.075	0.095	19.9	
	15408	41780	0.091	0.083	18.4	
0.6%	15209	42737	0.083	0.088	20.5	20.5 ± 2.0
	14188	42523	0.078	0.095	22.4	
	20365	41780	0.089	0.083	24.6	
1.5%	20744	42737	0.084	0.088	27.7	27.9 ± 3.4
	20580	42523	0.081	0.095	31.3	
	22879	41780	0.088	0.083	28.0	
3.0%	24379	42737	0.083	0.088	32.7	31.7 ± 3.3
	22883	42523	0.081	0.095	34.5	
	24851	41780	0.086	0.083	31.4	
6.0%	27052	42737	0.086	0.088	35.1	35.1 ± 3.8
	25244	42523	0.080	0.095	38.9	
	25812	41780	0.098	0.083	28.6	
9.0%	28478	42737	0.094	0.088	34.0	33.8 ± 5.1
	26863	42523	0.085	0.095	38.8	
	24824	41780	0.102	0.083	26.4	
27.0%	28612	42737	0.107	0.088	29.8	29.0 ± 2.4
	25636	42523	0.102	0.095	30.9	

Table S6. Quantum yield calculation table for **Phe2** with increasing amounts of **Py2**. Quinine sulfate in 0.5 M H_2SO_4 was used as a reference – Φ_R^{QS} = 0.546. Conditions: 10 °C/min cooling gradient, 3 µM **Phe2**, 10 mM sodium phosphate buffer pH 7.2, 10 mM NaCl and 20% EtOH.

	I_S	I_R	$A_{S\text{ - }320\text{ nm}}$	$A_{R\text{ - }320\text{ nm}}$	ϕ_S	ϕ_F
0.0%	8492	41780	0.086	0.083	10.7	11.8 ± 1.1
	8892	42737	0.084	0.088	11.8	
	8619	42523	0.082	0.095	12.9	
0.3%	9846	41780	0.083	0.083	12.8	14.1 ± 1.4
	10655	42737	0.086	0.088	13.9	
	10419	42523	0.082	0.095	15.5	
0.6%	11629	41780	0.084	0.083	14.9	16.2 ± 1.5
	12323	42737	0.088	0.088	15.8	
	12101	42523	0.083	0.095	17.8	
1.5%	16345	41780	0.082	0.083	21.7	22.8 ± 1.7
	17143	42737	0.088	0.088	21.9	
	17021	42523	0.084	0.095	24.8	
3.0%	20756	41780	0.084	0.083	26.7	28.3 ± 1.8
	22749	42737	0.091	0.088	28.0	
	21382	42523	0.087	0.095	30.2	
6.0%	24710	41780	0.082	0.083	32.4	34.6 ± 2.5
	28108	42737	0.101	0.088	34.1	
	26293	42523	0.106	0.095	37.3	
9.0%	26828	41780	0.099	0.083	29.3	33.8 ± 4.3
	30195	42737	0.098	0.088	34.4	
	28438	42523	0.092	0.095	37.8	
27.0%	28681	41780	0.106	0.083	29.1	32.0 ± 2.7
	33252	42737	0.115	0.088	32.4	
	30630	42523	0.109	0.095	34.6	

8. Light-Harvesting Spectroscopic Data

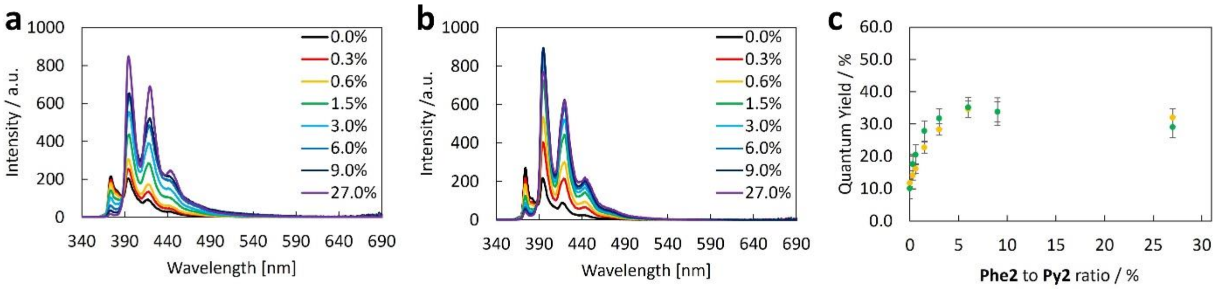

Figure S27 Fluorescence emission spectra of **Phe2** in the presence of increasing amounts of **Py2** (0 to 27%) with a cooling rate of (a) 0.5 °C/min and (b) 10 °C/min. (c) Quantum yield of both systems, in green at 0.5 °C/min and in yellow at 10 °C/min. Conditions: 3 µM **Phe2**, 10 mM sodium phosphate buffer pH 7.2, 10 mM NaCl and 20% EtOH, λ_{ex}: 320 nm.

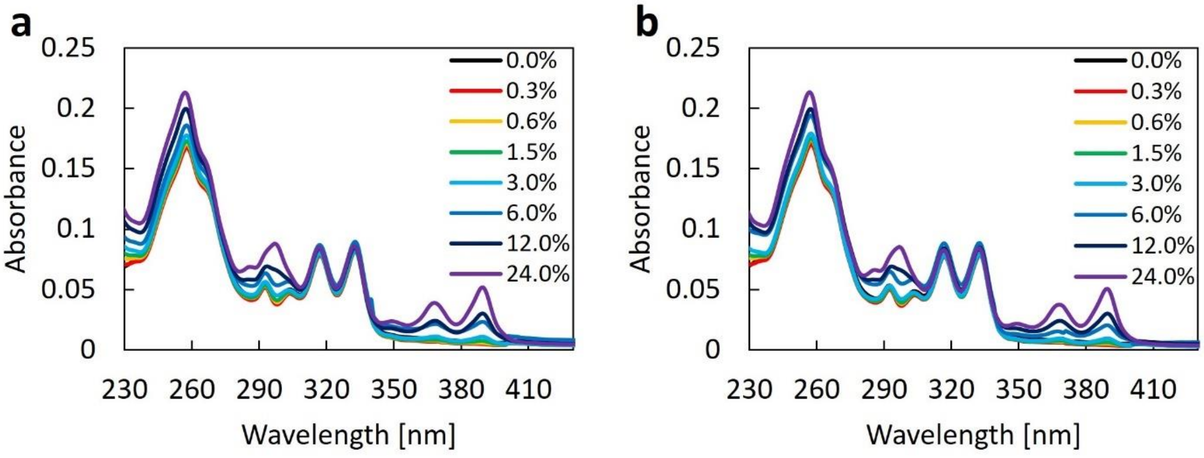

Figure S28 UV-Vis absorption spectra of **Phe1** with increasing amounts of **Py1** (0% to 24%). Conditions: 3 µM **Phe1**, 10 mM sodium phosphate buffer pH 7.2, 10 mM NaCl and 15% EtOH; cooling rate (a) 0.5 °C/min, (b) 10 °C/min.

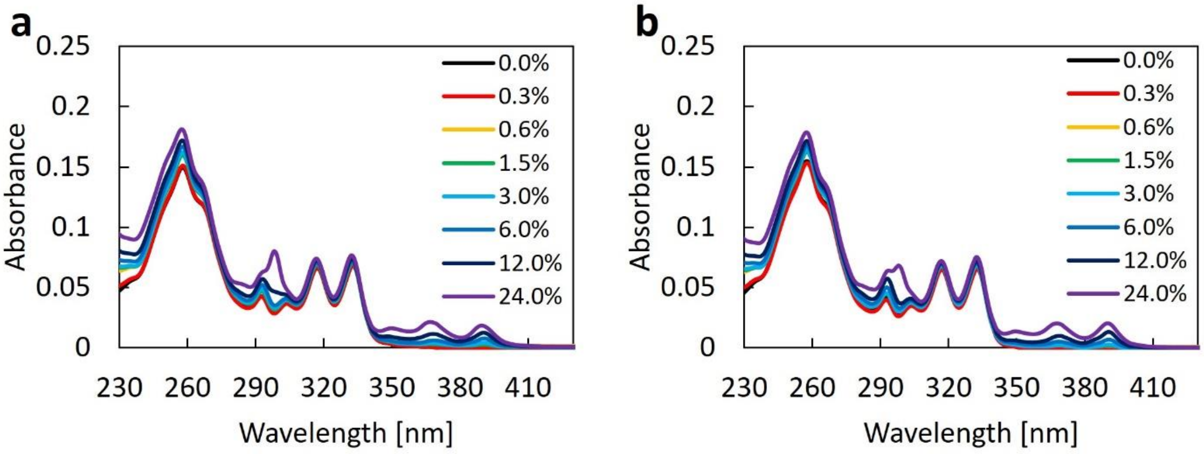

Figure S29 UV-Vis absorption spectra of **Phe1** with increasing amounts of **Py2** (0% to 24%). Conditions: 3 µM **Phe1**, 10 mM sodium phosphate buffer pH 7.2, 10 mM NaCl and 15% EtOH; cooling rate (a) 0.5 °C/min, (b) 10 °C/min.

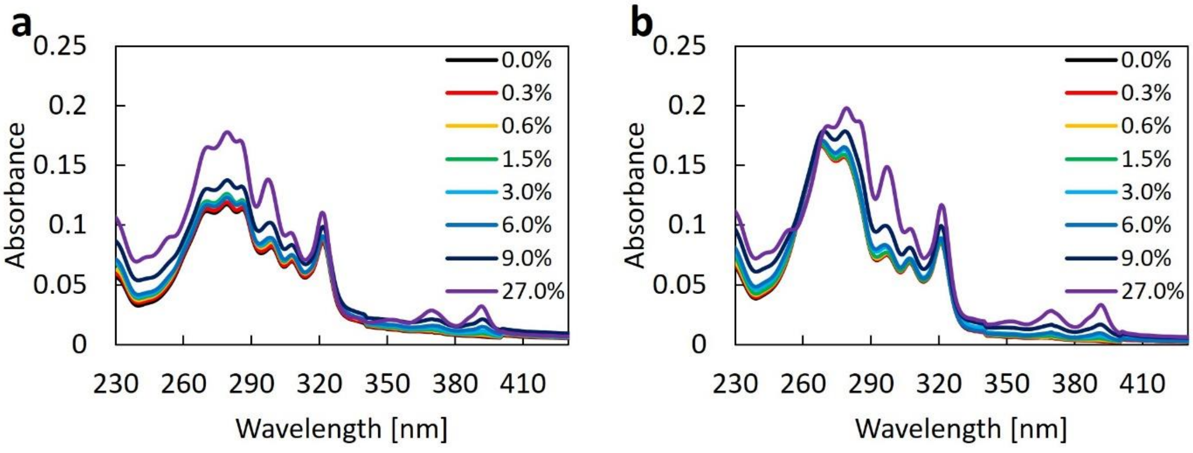

Figure S30 UV-Vis absorption spectra of **Phe2** with increasing amounts of **Py2** (0% to 27%). Conditions: 3 µM **Phe2**, 10 mM sodium phosphate buffer pH 7.2, 10 mM NaCl and 20% EtOH; cooling rate (a) 0.5 °C/min, (b) 10 °C/min.

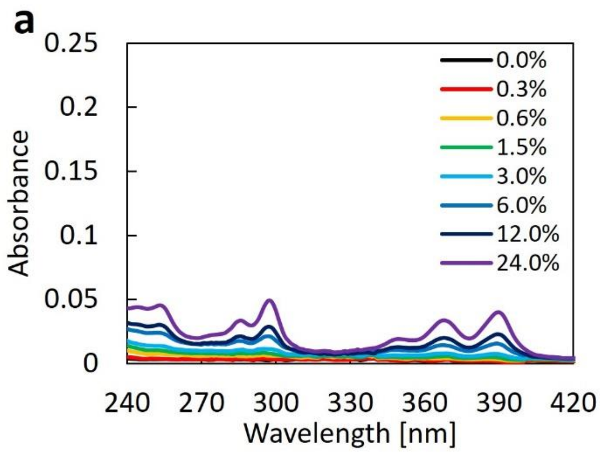

Figure S31 UV-Vis absorption spectra of the system in the absence of **Phe1** with increasing amounts of **Py1** (0% to 24%). Conditions: 10 mM sodium phosphate buffer pH 7.2, 10 mM NaCl and 15% EtOH; cooling rate (a) 0.5 °C/min, (b) 10 °C/min.

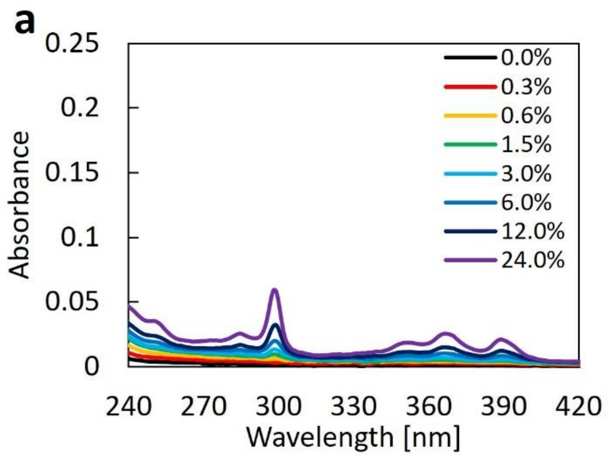

Figure S32 UV-Vis absorption spectra of the system in the absence of **Phe1** with increasing amounts of **Py2** (0% to 24%). Conditions: 10 mM sodium phosphate buffer pH 7.2, 10 mM NaCl and 15% EtOH; cooling rate (a) 0.5 °C/min, (b) 10 °C/min.

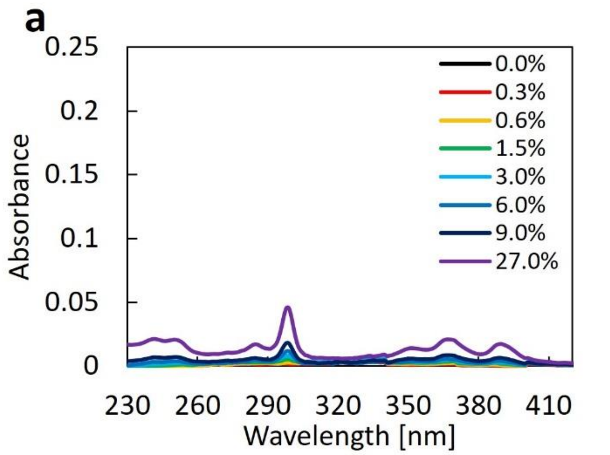

Figure S33 UV-Vis absorption spectra of the system in the absence of **Phe2** with increasing amounts of **Py2** (0% to 27%). Conditions: 10 mM sodium phosphate buffer pH 7.2, 10 mM NaCl and 20% EtOH; cooling rate (a) 0.5 °C/min, (b) 10 °C/min.

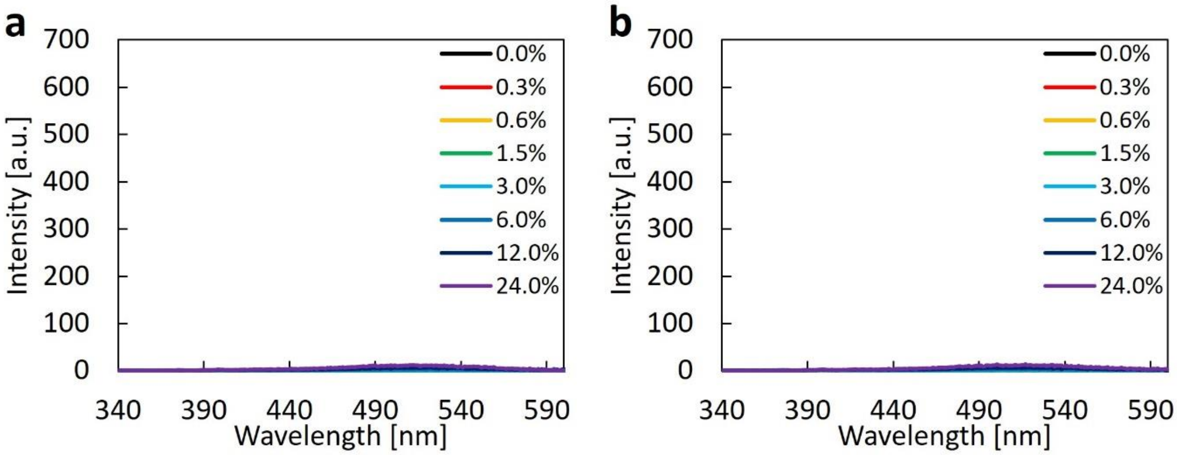

Figure S34 Fluorescence emission spectra of the system in the absence of **Phe1** with increasing amounts of **Py1** (0% to 24%). Conditions: 10 mM sodium phosphate buffer pH 7.2, 10 mM NaCl and 15% EtOH; λ_{ex}: 333 nm; cooling rate (a) 0.5 °C/min, (b) 10 °C/min.

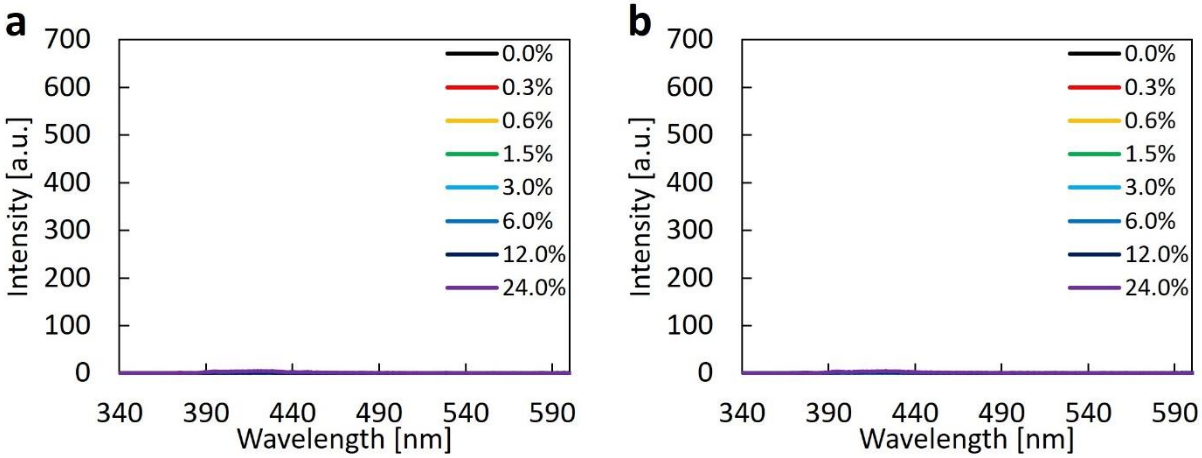

Figure S35 Fluorescence emission spectra of the system in the absence of **Phe1** with increasing amounts of **Py2** (0% to 24%). Conditions: 10 mM sodium phosphate buffer pH 7.2, 10 mM NaCl and 15% EtOH; λ_{ex}: 333 nm; cooling rate (a) 0.5 °C/min, (b) 10 °C/min.

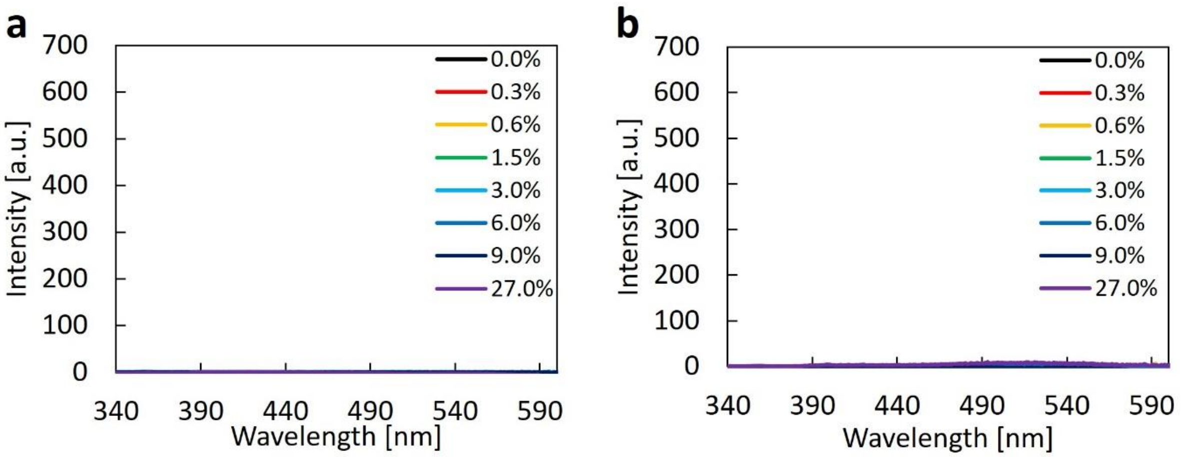

Figure S36 Fluorescence emission spectra of the system in the absence of **Phe2** with increasing amounts of **Py2** (0% to 24%). Conditions: 10 mM sodium phosphate buffer pH 7.2, 10 mM NaCl and 20% EtOH; λ_{ex}: 320 nm; cooling rate (a) 0.5 °C/min, (b) 10 °C/min.

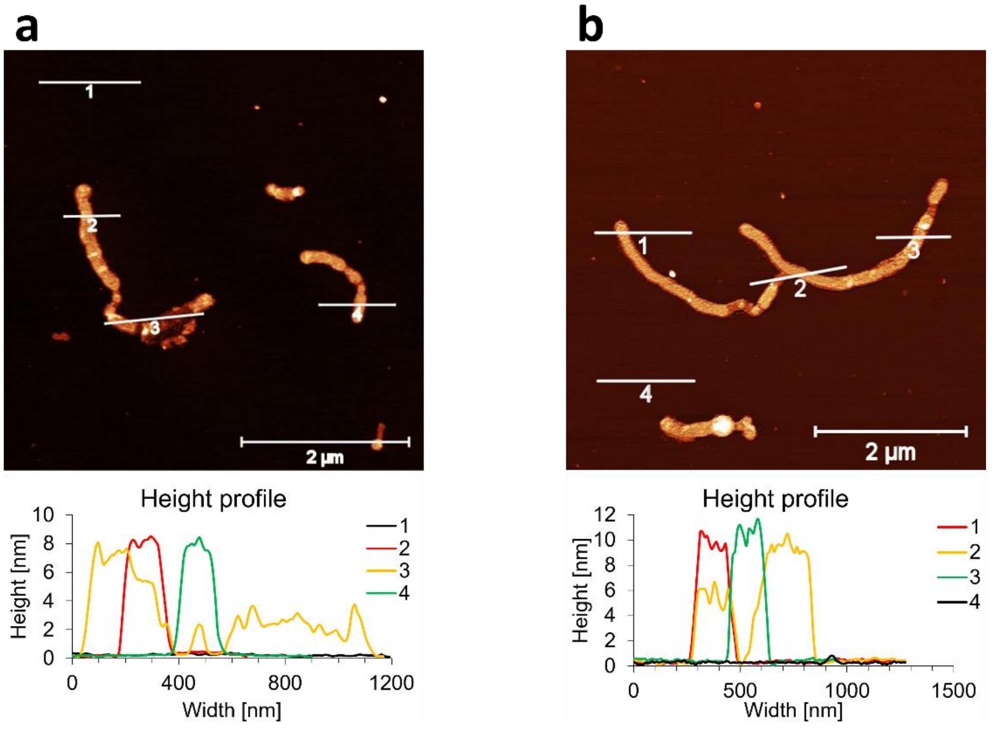

Figure S37 AFM images of the self-assembled **Phe1** (temperature gradient: 0.5 °C per min) on an APTES-modified mica with cross-sections in the presence of 6% of (a) **Py1** or (b) **Py2**. Conditions: 3 µM **Phe1**, 10 mM sodium phosphate buffer pH 7.2, 10 mM NaCl and 15% EtOH.

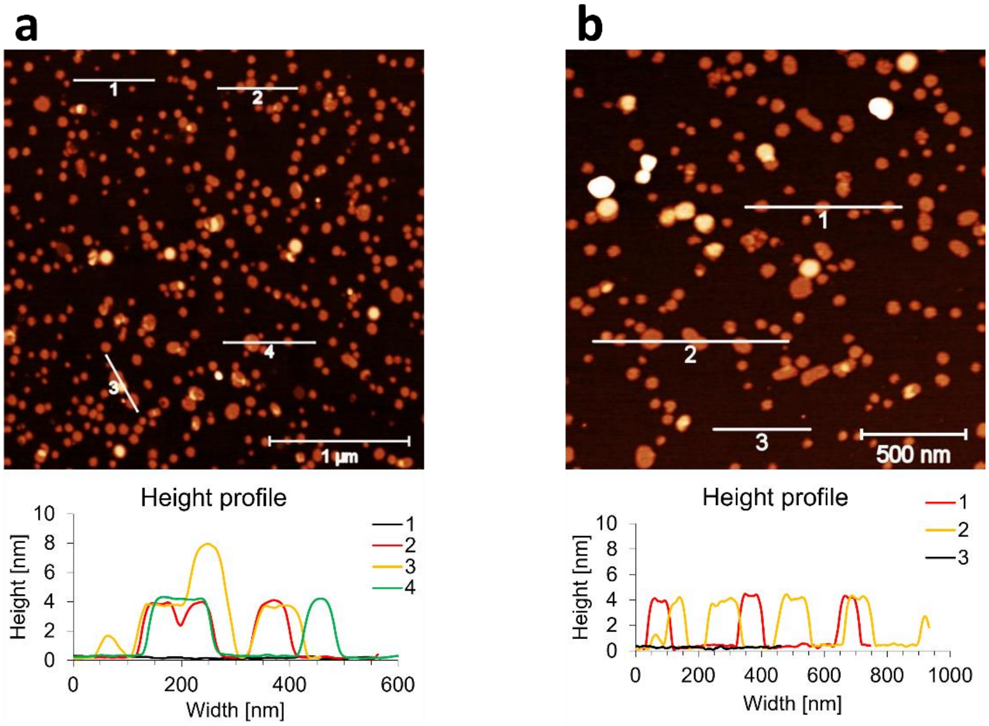

Figure S38 AFM images of the self-assembled **Phe1** (temperature gradient: 10 °C per min) on an APTES-modified mica with cross-sections in the presence of 6% of (a) **Py1** or (b) **Py2**. Conditions: 3 µM **Phe1**, 10 mM sodium phosphate buffer pH 7.2, 10 mM NaCl and 15% EtOH.

9. References

1 S. Rothenbühler, I. Iacovache, S. M. Langenegger, B. Zuber and R. Häner, *Nanoscale*, 2020, **12**, 21118–21123.

2 M. Linkert, C. T. Rueden, C. Allan, J.-M. Burel, W. Moore, A. Patterson, B. Loranger, J. Moore, C. Neves, D. MacDonald, A. Tarkowska, C. Sticco, E. Hill, M. Rossner, K. W. Eliceiri and J. R. Swedlow, *J. Cell Biol.*, 2010, **189**, 777–782.

3 J. Schindelin, I. Arganda-Carreras, E. Frise, V. Kaynig, M. Longair, T. Pietzsch, S. Preibisch, C. Rueden, S. Saalfeld, B. Schmid, J.-Y. Tinevez, D. J. White, V. Hartenstein, K. Eliceiri, P. Tomancak and A. Cardona, *Nat. Methods*, 2012, **9**, 676–682.

4 S. J. Kim, E.-K. Bang, H. J. Kwon, J. S. Shim and B. H. Kim, *ChemBioChem*, 2004, **5**, 1517–1522.
